# Genome sequencing of oomycete isolates from Chile supports the New Zealand origin of *Phytophthora kernoviae *and makes available the first *Nothophytophthora* sp. genome

**DOI:** 10.1111/mpp.12765

**Published:** 2018-12-05

**Authors:** David J. Studholme, Preeti Panda, Eugenio Sanfuentes Von Stowasser, Mariela González, Rowena Hill, Christine Sambles, Murray Grant, Nari M. Williams, Rebecca L. McDougal

**Affiliations:** ^1^ Biosciences, University of Exeter Stocker Road Exeter EX4 4QD UK; ^2^ Scion (New Zealand Forest Research Institute, Ltd.) Rotorua 3015 New Zealand; ^3^ Laboratorio de Patología Forestal, Facultad Ciencias Forestales y Centro de Biotecnología Universidad de Concepción Concepción 4070386 Chile; ^4^ Jodrell Laboratory Royal Botanic Gardens Kew TW9 3DS UK; ^5^ Life Sciences University of Warwick Coventry CV4 7AL UK

**Keywords:** *Drimys winteri*, *Eucalyptus nitens*, forest disease, hybrid, *Nothophytophthora*, oomycete, *Phytophthora*

## Abstract

Genome sequences were generated for six oomycete isolates collected from forests in Valdivia, Chile. Three of the isolates were identified morphologically as *Phytophthora kernoviae*, whereas two were similar to other clade 10 *Phytophthora* species. One isolate was tentatively identified as *Nothophytophthora valdiviana* based on nucleotide sequence similarity in the cytochrome oxidase 1 gene. This is the first genome sequence for this recently described genus. The genome assembly was more fragmented and contained many duplicated genes when compared with the other *Phytophthora* sequences. Comparative analyses were performed with genomic sequences of the *P. kernoviae* isolates from the UK and New Zealand. Although the potential New Zealand origin of *P. kernoviae *has been suggested, new isolations from Chile had cast doubt on this hypothesis. We present evidence supporting *P. kernoviae* as having originated in New Zealand. However, investigation of the diversity of oomycete species in Chile has been limited and warrants further exploration. We demonstrate the expediency of genomic analyses in determining phylogenetic relationships between isolates within new and often scantly represented taxonomic groups, such as *Phytophthora* clade 10 and *Nothophytophthora*. Data are available on GenBank via BioProject accession number PRJNA352331.

Pests and diseases, together with climate change, present the biggest threats to forest health (Ramsfield *et al.*, 2016; Trumbore *et al.*, 2015). The abundance and diversity of *Phytophthora* species known to be negatively impacting forest trees are increasing (Hansen, 2015; Scott and Williams, 2014; Studholme *et al.*, 2016). The knowledge of *Phytophthora* species diversity within forests is limited (Hansen *et al.*, 2012), although momentum is building to characterize these populations phylogenetically and to better understand their biology and impacts on forest health (Burgess *et al.*, 2017; Scarlett *et al.*, 2015). Tree diseases are likely to be ongoing and permanent features of the Anthropocene forest landscape as a result of human activities (Potter and Urquhart, 2017) with economic, landscape and societal impacts (Drake and Jones, 2017). For these reasons, it is essential that we understand the introduction pathways and how new pathogen populations emerge, and establish ways to mitigate the impacts of forest diseases.

*Phytophthora kernoviae* was first described in 2003, causing stem cankers on forest trees and foliar lesions on ornamentals in the UK (Brasier *et al.*, 2005). Subsequent studies discovered *P. kernoviae* in New Zealand, where it may have been present since at least the 1950s (Ramsfield *et al.*, 2007). In New Zealand, *P. kernoviae* is not considered to be a major pathogen of native flora, but has greater pathogenicity on exotic plant hosts (Gardner *et al.*, 2015). *Phytophthora kernoviae* has also been co‐isolated from needles exhibiting red needle cast in New Zealand’s *Pinus radiata* plantations, but not as frequently as *Phytophthora pluvialis* (Dick *et al.*, 2014), and not in association with substantial disease or mortality (Scott and Williams, 2014). The absence of symptoms on native host plants, and the long history of its presence, previously led to speculation that New Zealand may be the centre of origin for *P. kernoviae*.

*Phytophthora kernoviae* has been isolated from *Drimys winteri* (winter’s bark or canelo) in native forests in Chile (Sanfuentes *et al.*, 2016). Unlike the situation in New Zealand, *P. kernoviae* has not been detected in *P. radiata* plantations in Chile, in places in which severe attacks of *P. pinifolia* normally occur (E. Sanfuentes, personal information). Previously known only in Europe (UK) and New Zealand, the discovery of *P. kernoviae* on a third continent, and the common ancient Gondwanaland flora shared by New Zealand and Chile, raise questions as to its true place of origin. This might be resolved by the determination of phylogenetic relationships among isolates from each geographical location.

Genomes have been sequenced for isolates of *P. kernoviae* from New Zealand (Studholme *et al.*, 2016) and the UK (Feau *et al.*, 2016; Sambles *et al.*, 2015). Here, we report the first genome sequences for isolates of *P. kernoviae* from Chile (Table 1), the only other geographical location in which this species has been reported so far. Furthermore, we present the genome sequences of two unidentified isolates that belong to the *Phytophthora* internal transcribed spacer (ITS) clade 10 (Cooke *et al.*, 2000), and report the first genome sequence for a member of *Nothophytophthora*, a newly described sister genus to *Phytophthora* (Jung *et al.*, 2017). This *Nothophytophthora* isolate was collected from infected *Eucalyptus nitens* foliage, indicating the pathogenic potential for this genus, revealing a hitherto unrecognized potential threat to forest trees. The genetic diversity observed in these genome sequences demonstrates that the biodiversity of oomycetes in Chilean forests warrants further investigation. With increased efforts to understand the diversity and distribution of potential pathogens, there is a need to quickly establish the identity, diversity and distribution of newly described isolates. The main objective of this study was to determine the relationship of newly discovered Chilean *P. kernoviae* to isolates from Europe and New Zealand. A second objective was to characterize other *Phytophthora*‐like isolates from Chilean forests and their taxonomic and phylogenetic positions within the oomycetes to obtain a better understanding of the diversity and biosecurity implications of the species.

**Table 1:** Isolates used in this study and summary statistics for genome sequence data.

Isolate	Year of isolation	Source/host	Region	Accession numbers: GenBank and SRA	Total size (bp)	Coverage depth	Number of scaffolds	Scaffold N50 (bp)	Number of annotated gene models	Repeat content (%)
Chile 1	2014	Baited leaf litter from *Drimys winteri* forest	Llancahue, Valdivia, Chile	MBAB00000000, SRR4865694	38 111 184	50×	2422	62 563	9914	14.27
Chile 2	2012	Baited leaf litter from *Drimys winteri* forest	Llancahue, Valdivia, Chile	MAYM00000000, SRR4865680	38 203 779	92×	2393	64 455	9922	13.88
Chile 4	2012	Baited leaf litter from *Drimys winteri* forest	Llancahue, Valdivia, Chile	MBDN00000000, SRR4865689	37 458 212	50×	2034	64 544	9877	11.18
Chile 5	2014	*Eucalyptus nitens* (infected foliage)	Ciruelos, Valdivia, Chile	MBAC00000000, SRR4865670	84 445 542	40×	11 901	15 776	11 952	16.48
Chile 6	2014	Baited leaf litter from *Drimys winteri* forest (relict inside *Eucalyptus nitens* forest)	Ciruelos, Valdivia, Chile	MBDO00000000, SRR4865676	36 780 765	57×	1910	63 953	10 093	10.72
Chile 7	2014	Baited soil from mixed forest (*Nothofagus* forest and other species)	Caramávida, Los Álamos, Chile	MBAD00000000, SRR4865684	37 002 228	30×	2830	33 020	10 129	12.11

*Phytophthora* isolates were obtained from soil, leaf litter and infected *E. nitens* foliage samples collected from three locations in Chile (Table 1) using standard baiting and isolation techniques (Erwin and Ribero, 1996). Preliminary identification of each isolate was performed using DNA sequences of ITS‐1 and ITS‐4 (White *et al*., 1990 as described in Jung *et al.*. (2017). For genome sequencing, DNA was extracted from mycelium using a GeneJET DNA purification kit (Thermo Fisher), and its quality was assessed using a Qubit fluorometer (Thermo Fisher, Waltham, MA, USA.) and agarose gel electrophoresis. Genomic DNA from each isolate was sequenced using an Illumina HiSeq 2500 (San Diego, CA, USA) to generate 295‐bp paired‐end reads. A *de novo* assembly and scaffolding were performed using SPAdes v3.8.1 (Bankevich *et al.*, 2012) after first removing poor‐quality data and adaptor sequences using TrimGalore (https://www.bioinformatics.babraham.ac.uk/projects/trim_galore/). Genomes were annotated with predicted genes identified using the MAKER pipeline v2.31.10 (Cantarel *et al.*, 2008), incorporating *ab initio* gene prediction based on AUGUSTUS v3.1.0 trained on *P. kernoviae* genes; full details are provided via the MAKER configuration files in Text S1 (see Supporting Information). Annotated genome assemblies and raw sequence data are available via GenBank (Table 1). The assembly statistics for the genome sequences presented are comparable with those published previously (Feau *et al.*, 2016; Sambles *et al.*, 2015; Studholme *et al.*, 2016), with the exception of Chile 5. This genome assembly had a larger number of scaffolds and appeared to be much larger in total length (Table 1). The completeness of the genome assemblies was assessed using BUSCO v2 (Benchmarking Universal Single‐Copy Orthologs) (Simao *et al.*, 2015) to check the presence of conserved single‐copy orthologous genes commonly conserved across the Alveolata–Stramenopile lineage. This analysis was performed on both the genome sequences and predicted proteomes (Table 2). Completeness for genes from Chile 1, Chile 2, Chile 4, Chile 6 and Chile 7 (95.7%–97.9%) was similar to that of recently published *P*. *kernoviae* genomes (97.4%–97.9%) (Feau *et al.*, 2016; Sambles *et al.*, 2015; Studholme *et al.*, 2016) (Table 2). The Chile 5 assembly was less complete (87.6%) and had a considerably higher number of duplicated genes (164) compared with the other genomes, which had less than four duplicated genes. In addition, the Chile 5 assembly was missing more genes (25) than the other genomes (Table 2). This pattern was also reflected in the predicted proteomes and may indicate that Chile 5 is a hybrid. Similar observations have been made previously with BUSCO analysis of predicted genes by Feau *et al. *(2016) for the genome sequence of *P. alni* sp. *alni*, a well‐characterized hybrid *Phytophthora* species. Jung *et al. *(2017) noted the presence of heterozygous nucleotides within several gene regions in their *Nothophytophthora* isolates, and suggested the need for further investigation of hybridization or ploidy variation.

**Table 2:** BUSCO (Benchmarking Universal Single‐Copy Orthologs) analysis for annotated genes and proteins.

	BUSCO analysis of genome sequences					BUSCO analysis of predicted protein sequences					
Isolate	Complete genes (% complete)*	Complete and single‐copy proteins	Complete and duplicated proteins	Fragmented genes*	Missing genes*	Complete proteins (% complete)*	Complete and single‐copy proteins	Complete and duplicated proteins	Fragmented proteins	Missing proteins	Reference†
Chile 1	229 (97.9)	229	0	3	2	205 (87.6)	205	0	8	21	This study
Chile 2	228 (97.4)	228	0	3	3	205 (87.6)	205	0	8	21	This study
Chile 4	228 (97.4)	228	0	3	3	204 (87.6)	204	0	7	23	This study
Chile 5	205 (87.6)	41	164	4	25	163 (69.6)	41	122	9	62	This study
Chile 6	228 (97.4)	227	1	4	2	203 (86.8)	203	0	7	24	This study
Chile 7	224 (95.7)	223	1	4	6	198 (84.6)	198	0	8	28	This study
00629/1	228 (97.4)	228	0	2	4	–‡	–	–	–	–	Sambles *et al*. (2015)
00238/432	229 (97.9)	229	0	2	3	207	207	0	7	20	Sambles *et al.* (2015)
00844/4	229 (97.9)	229	0	2	3	–‡	–	–	–	–	Sambles *et al.* (2015)
CBS 122049	213 (91.1)	207	6	5	16	–‡	–	–	–	–	Feau *et al.* (2016)
NZFS 2646	230 (98.3)	230	0	2	2	208	208	0	6	20	Studholme *et al.* (2016)
NZFS 3630	230 (98.3)	230	0	2	2	207	207	0	7	20	Studholme *et al.* (2016)

*BUSCO coverage when tested with Alveolata–Stramenopile gene set (*n* = 234 genes) (Simao *et al.*, 2015)

^†^Reference for dataset description and BUSCO analysis with annotated genes. BUSCO analysis with annotated proteins performed in this study.

^‡^–, not determined. Genomes from strains 00629/1 and 00844/4 are almost identical to that of 00238/432 (Sambles *et al.*, 2015), and strain CBS 122049 is also very similar to the other UK strains; hence, 00238/432 was used solely to represent the UK strains.

To initially identify the species of the isolates, we extracted the cytochrome oxidase I (COI) gene sequences from the genome assemblies and performed BLASTN (Altschul *et al.*, 1990) similarity searches against the National Center for Biotechnology Information non‐redundant (NCBI NR) database. The COI sequences from Chile 1, Chile 2 and Chile 4 were 99% identical to those of other *P. kernoviae *sequences. Sequences from Chile 6 and Chile 7 also showed close matches to *P. kernoviae*, but at 97% identity (data not shown). Interestingly, the COI sequence of Chile 5 exhibited 100% identity to that of *Nothophytophthora* *valdiviana* CL322 (GenBank: KY788506) (data not shown).

To more robustly assess the relationships between the genomes of the sequenced isolates, the Reference Sequence Alignment‐based Phylogeny Builder (REALPHY) tool was used to reconstruct the phylogenies from raw genome sequencing reads (Bertels *et al.*, 2014). Sequencing reads from the six Chilean genomes were aligned with those of the other available *P*.* kernoviae* isolates from the UK and New Zealand (Sambles *et al.*, 2015; Studholme *et al.*, 2016). Chile 1, Chile 2 and Chile 4 clustered adjacent to the *P. kernoviae* isolates from New Zealand and the UK (Fig. 1), consistent with the phylogenies constructed using COI (Text S2; Fig. S1, see Supporting Information), indicating that these isolates were *P. kernoviae*. Chile 6 and Chile 7 appeared divergent to the other Chilean isolates (Fig. 1), clustering separately from *P. kernoviae* and other clade 10 *Phytophthora* species. Chile 5 clustered separately to all *Phytophthora* genomes in each constructed phylogeny, but clustered with *Nothophytophthora* in the COI alignment (Fig. S1).

**Figure 1:**
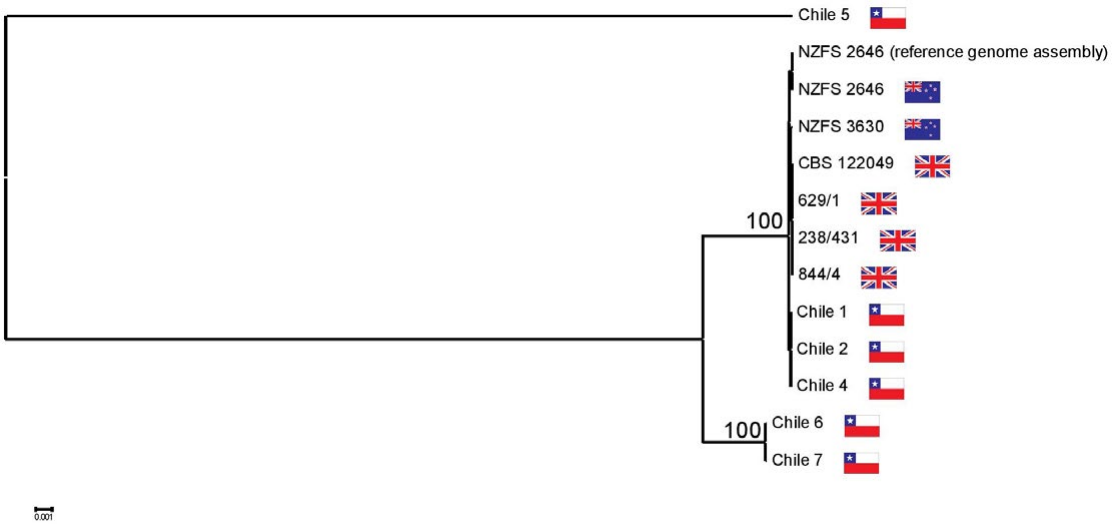
Phylogenetic positions of Chilean *Phytophthora* isolates based on whole‐genome sequencing. Geographical locations of isolation are indicated by country flags (Chile, New Zealand and UK). Genomic sequence reads and the reference genome assembly for New Zealand and UK isolates were obtained from previous studies (Sambles *et al.*, 2015; Studholme *et al.*, 2016). The phylogenetic tree was generated using the Reference Sequence Alignment‐based Phylogeny Builder (REALPHY) tool (Bertels *et al.*, 2014) with RAxML 8.2.9 (Stamatakis, 2014) as its tree builder and Bowtie 2.3.0 for alignment of the genomic sequence reads against the assembled NZFS 2646 reference genome sequence. Bootstrap values are given as percentages of 500 trials. [Colour figure can be viewed at wileyonlinelibrary.com]

To complement the REALPHY‐based phylogenetic analysis, isolates were clustered according to the sequence similarity of their genome assemblies by pairwise genome alignments using the dnadiff tool in the software package MUMmer v3.23 (Kurtz *et al*., 2004). Average nucleotide identities (ANIs) of one‐to‐one alignments were obtained from dnadiff, and a heatmap of the ANI similarity matrix was generated using the BIONJ clustering method (Gascuel, 1997). This analysis supported the REALPHY results, with nucleotide sequence identities among all pairs of New Zealand and UK *P. kernoviae* isolates and Chilean isolates 1, 2 and 4 being greater than 99%, whereas Chile 6 and Chile 7 showed 97% identity with *P*. *kernoviae. *Furthermore, Chile 5 shared only 84% identity with the *P. kernoviae* isolates (Fig. 2), suggesting more distant relationships between these groups. Further support for the genetic diversity of New Zealand isolates was observed using the detected single nucleotide polymorphisms (SNPs) concatenated and aligned for a splits tree analysis (Text S2; Fig. S2, see Supporting Information). Genetic variation between the genomes of the New Zealand isolates was found to be greater than that for the UK or Chilean *P. kernoviae* isolates. Although this analysis would benefit from a greater number of genomes for comparison, it provides early insights suggesting that New Zealand may have greater genetic diversity and be the origin of this species.

**Figure 2:**
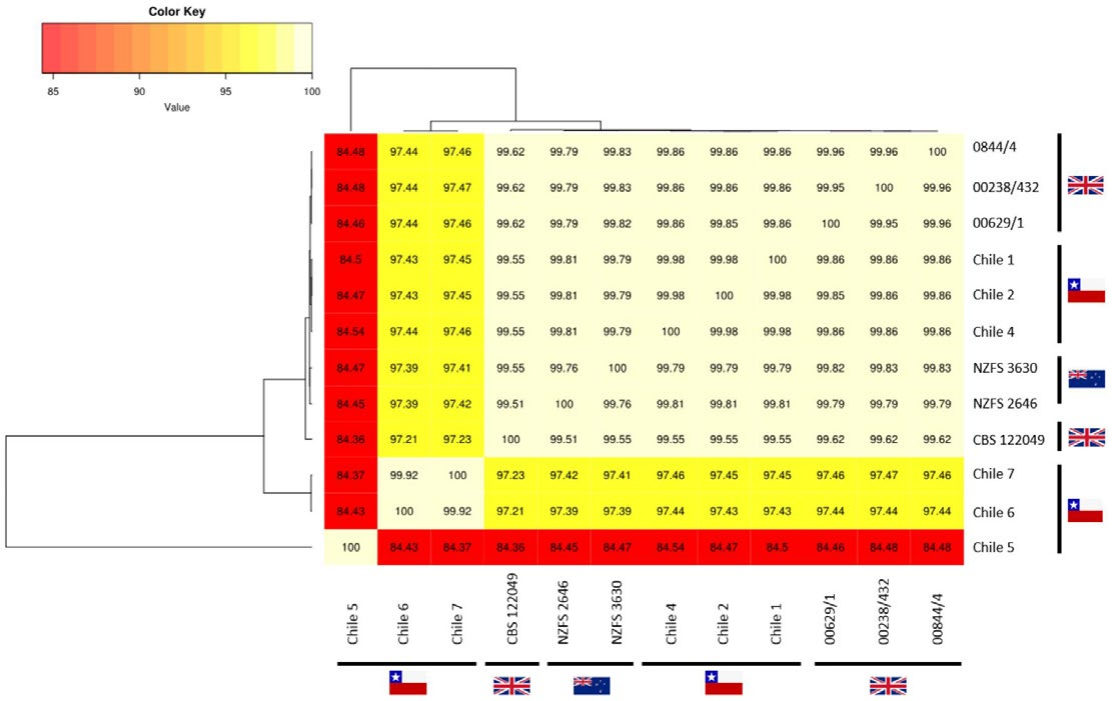
Average nucleotide identity matrix of *Phytophthora* sequence identities based on alignments of whole assembled genomes. The colour key represents the pairwise percentage similarity of alignment results from the dnadiff tool in MUMmer v3.23 (Kurtz *et al*., 2004). [Colour figure can be viewed at wileyonlinelibrary.com]

The interspersed repeats within the genomes of *P. kernoviae *isolates were determined by RepeatMasker v4.0.7 (Smit *et al*., 2015) using a combined database of DFam_Consensus v2017‐01‐27 (Wheeler *et al*., 2013) and RepBase Phytophthora library v2017‐01‐27 (Jurka *et al*., 2005). This revealed that the content of repetitive elements for the New Zealand strains was approximately 8.6% each, whereas the Chilean and UK isolates ranged from 10.7% to 16.48% and 5.98% to 7.48%, respectively (Table S1, see Supporting Information). Overall, these results are lower than those for previously reported genomes of *Phytophthora* species, such as *P. cactorum* (46.7%) (Yang *et al*., 2018), *P. ramorum* (28%), *P. sojae* (38%) and *P. infestans* (74%) (Haas *et al.*, 2009), but may simply reflect differences in sequencing and assembly methods, with redundant sequences being collapsed to single contigs. Of all the genomes analysed in this study, Chile 5 showed the greatest overall repetitive sequence content.

Genes encoding candidate RxLR and crinkler (CRN) effectors were predicted from the *P. kernoviae *genomes by searching for the RxLR‐EER and LFLAK‐HVLV motifs from amino acid sequences (translated from all of the open reading frames in a genome) using regular expression and homology search mechanisms based on hidden Markov models (HMMs) (Eddy, 1998; Tabima and Grunwald, 2018). Elicitins were predicted on the annotated gene models based on HMM models built from lists of known elicitins on UniProt (The UniProt Consortium, 2015) and established models of primary elicitin structures (Jiang *et al*., 2006). The candidate effector proteins were passed through the SignalP v3.0 (Dyrløv Bendtsen *et al*., 2004) program, and those with a predicted signal peptide were considered as candidate effectors. The numbers of predicted RxLR, CRN and elicitin effectors were similar for all *P. kernoviae* isolates, with the Chilean genomes showing slightly higher numbers of RxLRs compared with the other *P. kernoviae* genomes. By stark contrast, the genome from isolate Chile 5 revealed only five RxLR proteins compared with all other genomes which contained 116–159 RxLR proteins, and a much higher number of elicitins (88) compared with the others (<21) (Fig. S3, see Supporting Information). These results are consistent with the taxonomic and phylogenetic distinctness of Chile 5 compared with all other Chilean isolates, and highlights the diversity of virulence effectors and genetic diversity to be found among Chilean *Phytophthora* populations.

The extent of heterozygosity was estimated in each genome by aligning genomic sequence reads against the NZFS 2646 reference genome assembly. This approach assumes that, in shotgun sequencing of a diploid genome, sequence reads are drawn randomly from each chromosome, giving an estimate of heterozygosity across the genome (Turner *et al.*, 2017). Figure 3 shows the frequency of distributions for the most abundant and second most abundant base at each position in the genome. A highly homozygous genome would be expected to display a single peak close to 100% abundance for the most common base and a single peak close to zero for the second most abundant, with heterozygous sites contributing to a second peak close to 50% abundance representing an overlap in the most common and second most abundant base. The resulting plots of the estimated allele frequency show a pronounced peak close to 50% for the New Zealand isolate NZFS 3630 and, to a lesser extent, for NZFS 2646 and the UK isolates (Fig. 3). Given that *P. kernoviae* is homothallic (Brasier *et al.*, 2005), low levels of heterozygosity amongst most isolates could be explained by multiple generations of inbreeding (Goodwin, 1997), which would be consistent with a single introduction of a founder population with narrow genetic diversity. In contrast, a relatively high degree of heterozygosity (as observed for NZFS 3630) would be expected in individuals sampled from a genetically diverse and frequently outcrossing population, as would be expected at the centre of origin. Another possibility is that isolate NZFS 3630 is drawn from a population of individuals which reproduce exclusively by non‐sexual vegetative means. Although heterothallism has not yet been demonstrated in *P. kernoviae*, it is a possibility and cannot be excluded.

**Figure 3:**
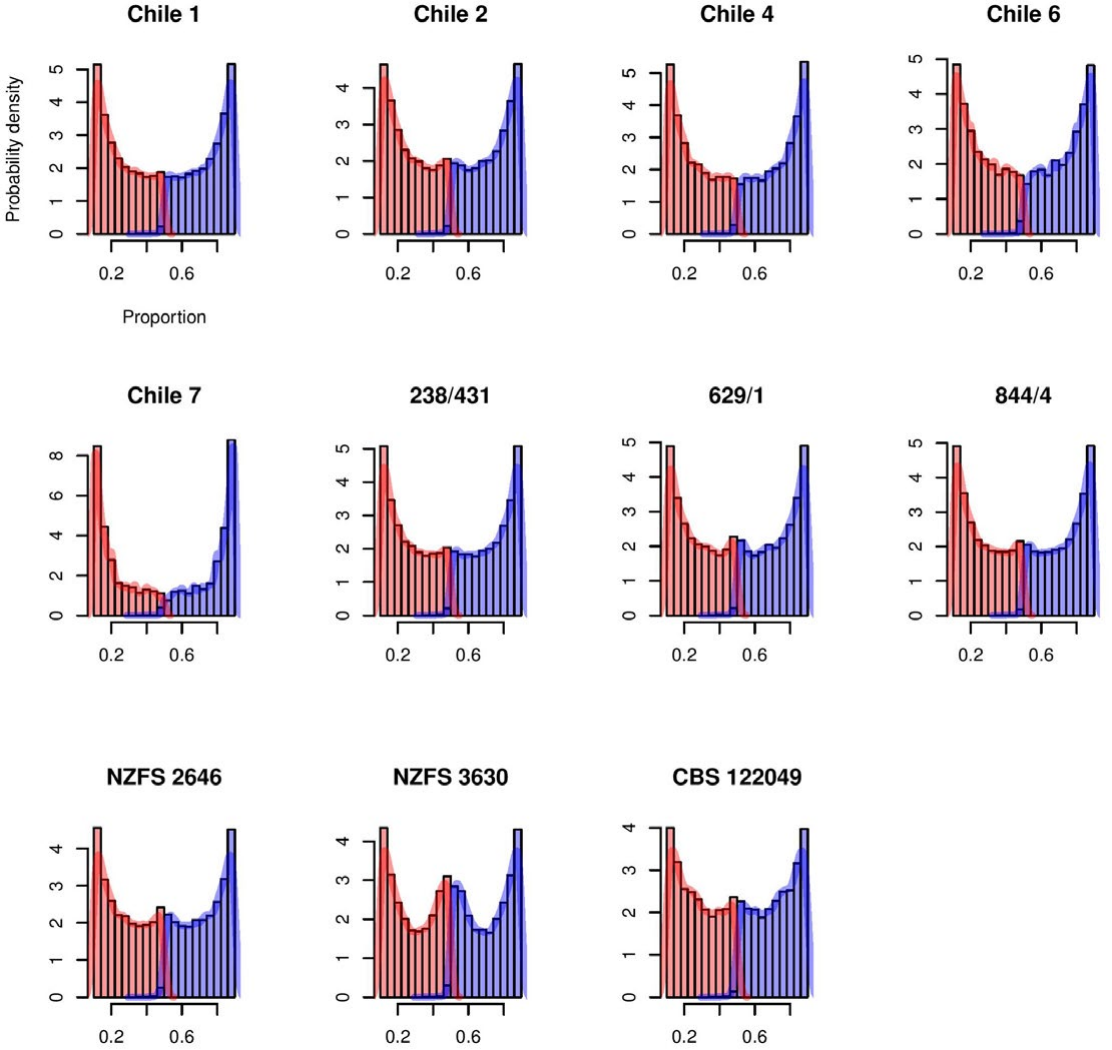
Frequency distributions of relative abundances of major alleles over all genomic sites in sequenced *Phytophthora kernoviae* genomes. For each isolate, sequence reads were aligned against the appropriate genome assembly using BWA‐mem (Li and Dublin, 2009; Li, 2013). The histogram shows the frequency densities for the relative abundance of the most common nucleotide in blue and the second most abundant nucleotide in red. The histograms have been cropped at 0.1 and 0.9 for clarity. [Colour figure can be viewed at wileyonlinelibrary.com]

Gondwanan origins have been investigated for several plant pathogens, including *Phytophthora cinnamomi* (Arentz, 2017). The potential Gondwanan ancestry of the isolates in this study remains plausible and is in agreement with the common ancestral plant diversity of both Chile and New Zealand (McCarthy *et al.*, 2007; Segovia *et al.*, 2015). Our data are consistent with previous studies proposing that *P. kernoviae *in the UK is of New Zealand origin; Fig. 1 shows the UK isolates forming a single narrow lineage within the radiation of diversity found in New Zealand. Chilean isolates 1, 2 and 4 represent an outgroup, not originating within the breadth of diversity found in New Zealand. Such a pattern is consistent with Chilean and New Zealand populations both being derived from an ancient pan‐Gondwanan population. Within New Zealand and Chile, the subtle and intermittent expression of disease symptoms on native plant hosts suggests that *P*. *kernoviae* populations have co‐evolved with plant hosts in both their respective ecosystems, reflecting the similarities in plant diversity and climate between the two countries. However, genotyping of further isolates sampling the genetic diversity in New Zealand and South America is necessary to test this hypothesis. Molecular clock models may infer the timing of the last common ancestor of New Zealand and Chilean populations. Previous attempts to date events in oomycete evolution have estimated the origin of the genus *Phytophthora* at less than 50 million years ago (Matari and Blair, 2014), implying that speciation in *Phytophthora* took place after the breakup of Gondwana about 180 million years ago. For greater confidence, molecular clock studies of the *Phytophthora* genus and Peronosporales would benefit from analyses across a much greater number of isolates, especially with the inclusion of basal clade 10 *Phytophthora* and *Halophytophthora* isolates.

Current phylogenies place *P. kernoviae *in clade 10 (Cooke *et al.*, 2000) of the genus, together with four other species: *Phytophthora boehmeriae* K. Sawada (Erwin and Robeiro, 1996), *Phytophthora gallica *T. Jung & J. Nechwatal (Jung and Nechwatal, 2008), *Phytophthora morindae* Z.G. Abad & S.C. Nelson (Nelson and Abad, 2017) and *Phytophthora intercalaris* (Yang *et al.*, 2015). *Phytophthora* *gallica *has been recovered from the rhizosphere of declining oak in France and Germany, and identified as a moderately aggressive pathogen, but of lower impact than other pathogens found within the same system (Jung and Nechwatal, 2008). In contrast, *P. boehmeriae *has long been associated with leaf, boll and root rot of a broad range of host species dating back to 1927 (Erwin and Robeiro, 1996). The newly described species, *P. morindae,* causes foliar and fruit rot disease of Indian Mulberry (*Morinda citrifolia* L. var. *citrifolia*) (Nelson and Abad, 2017), whereas *P. intercalaris* has frequently been isolated in stream baiting surveys of eastern USA, but has yet to be associated with disease symptoms on any plant host (Yang *et al.*, 2015). The increasing numbers of pathogens being assigned to *Phytophthora* clade 10, together with the known pathology of most of these species, demonstrate the potential for pathogens from this clade to cause significant disease on a range of hosts in suitable climatic conditions. The phylogenetic diversity amongst the few isolates presented here suggests that a considerable level of taxonomic diversity within clade 10 remains undescribed with implications for the emergence of new pathogens. Chilean isolates 5, 6 and 7 are clearly distinct, with Chile 5 probably being *Nothophytophthora* *valdiviana*, and Chile 6 and Chile 7 representing another separate lineage and possibly distinct species within the clade 10 *Phytophthora* species that may be unique to South America. The *Phytophthora* isolates studied here were all collected within a single region of Chile; a broader analysis of isolates from regions of a comparable climatic range to the temperate areas of New Zealand in which *P. kernoviae* has been isolated would be of considerable benefit.

The genome sequences presented here provide a resource for the development of genetic markers (e.g. SNPs and short repeats) that could be used for the assessment of population diversity (Brar *et al.*, 2018). This study also highlights the power of whole‐genome sequencing as a tool for the identification of *Phytophthora* species, including potential hybrids and yet‐to‐be described species.

## Supporting information


**Fig. S1** Relationships of Chilean *Phytophthora* isolates based on cytochrome oxidase I DNA sequences. *Phytophthora* isolates from Chile are indicated with an asterisk. IMI393172 is the *P. kernoviae* Holotype (CBS website). GenBank accession numbers are given for sequences from previous studies. The DNA sequences were aligned using the MAFFTT plugin in Geneious (v10.2.2) and the phylogenetic tree was constructed using the RAxML plugin and edited in FigTree V1.4.3 (http://tree.bio.ed.ac.uk/software/figtree/).

**Fig. S2** The relationship between eleven strains of *P. kernoviae* (from UK; n=4, NZ; n=2, and Chile; n=5) and closely related isolates (Chile 6 and 7 from Chile). SNPs detected from across the entire genomes were concatenated, aligned and a tree constructed using SplitsTree (Huson & Bryant, 2006). A; full Splitstree depicting relationships of all genomes, B; inset from A showing detailed relationships of *P. kernoviae* isolates. Chile 5 was not included in this analysis.

**Fig. S3** Numbers of predicted RxLR, CRN and elicitin genes from *Phytophthora* genomes. Elicitin gene numbers were not predicted from 0069/1, 00844/4 or CBS 122049. Genomes from strains 0069/1 and 00844/4 show a very high level of identity to that of 00238/432 (Studholme *et al.* unpublished), and strain CBS 122049 is also very similar to the other UK strains, hence 00238/432 was used solely to represent the UK strains.

**Table S1** Repeat Content of Chilean *Phytophthora kernoviae* and *Nothophytophthora* genomes.

**Text S1** Configuration files for MAKER genome annotation.

**Text S2** Supplementary methods. (a) Phylogenetic analysis of COI region. (b) Analysis of genetic relationships using genome‐wide SNPs and SplitsTree.
