# Supplementary figures and images for: Genome sequencing of oomycete isolates from Chile supports the New Zealand origin of Phytophthora kernoviae and makes available the first Nothophytophthora sp. genome
Source: Mol Plant Pathol. 2018 Dec 5;20(3):423–31. doi: 10.1111/mpp.12765 (PMC6637878; doi:10.1111/mpp.12765)

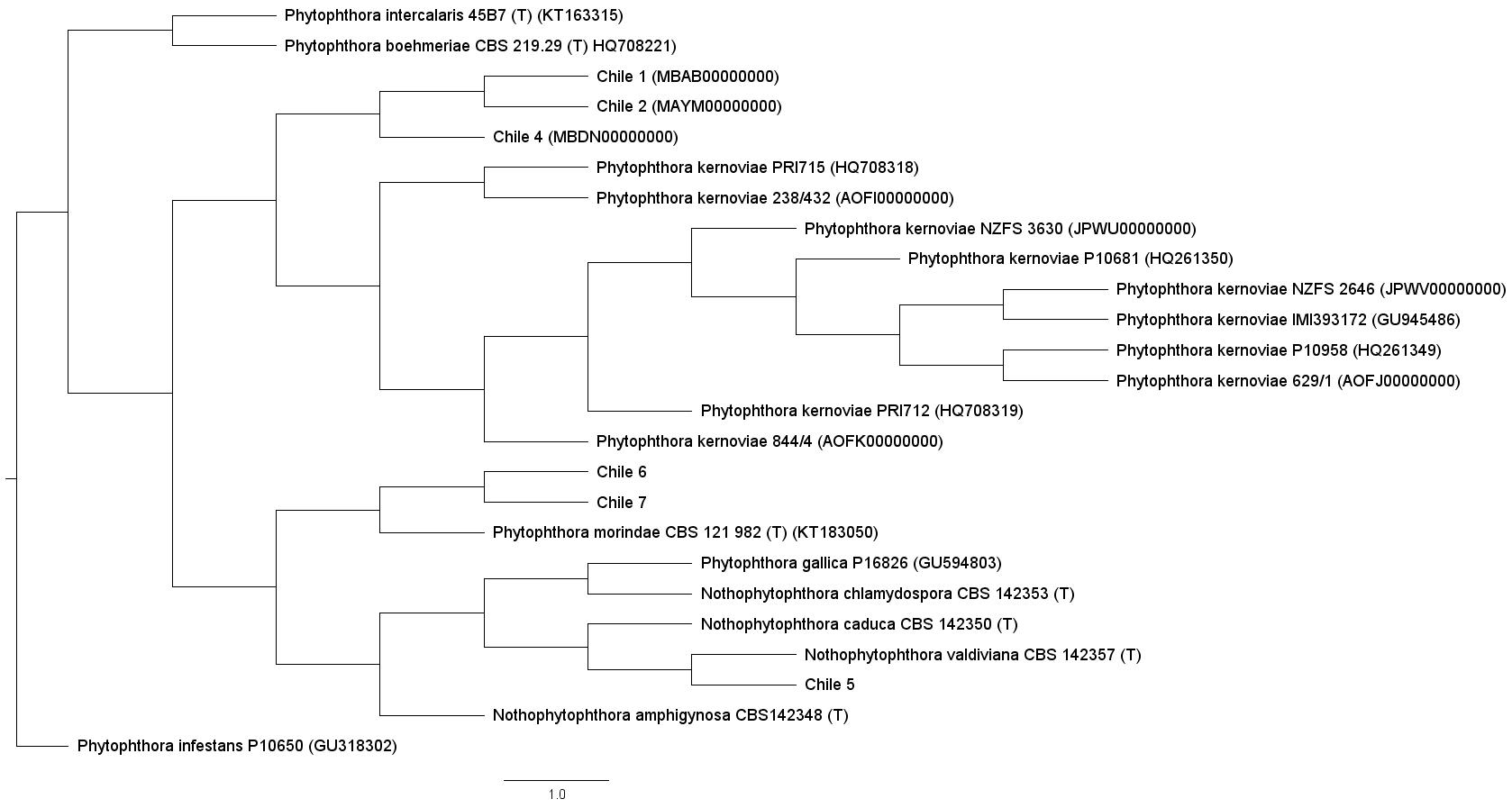

Supplement: Supplementary file 1 — Fig. S1 Relationships of Chilean Phytophthora isolates based on cytochrome oxidase I DNA sequences. Phytophthora isolates from Chile are indicated with an asterisk. IMI393172 is the P. kernoviae Holotype (CBS website). GenBank accession numbers are given for sequences from previous studies. The DNA sequences were aligned using the MAFFTT plugin in Geneious (v10.2.2) and the phylogenetic tree was constructed using the RAxML plugin and edited in FigTree V1.4.3 (http://tree.bio.ed.ac.uk/software/figtree/). [file MPP-20-423-s001.jpg]

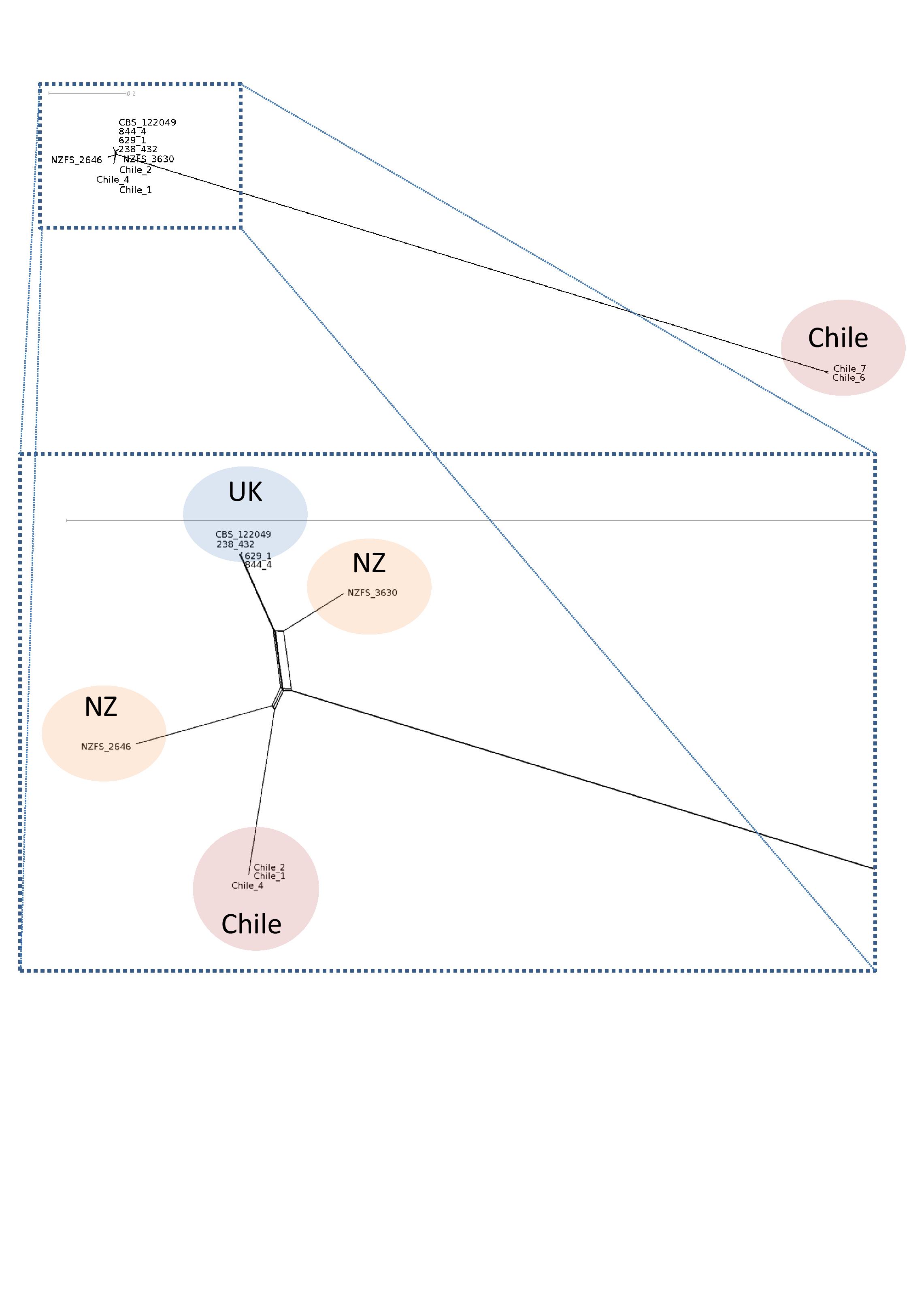

Supplement: Supplementary file 2 — Fig. S2 The relationship between eleven strains of P. kernoviae (from UK; n=4, NZ; n=2, and Chile; n=5) and closely related isolates (Chile 6 and 7 from Chile). SNPs detected from across the entire genomes were concatenated, aligned and a tree constructed using SplitsTree (Huson & Bryant, 2006). A; full Splitstree depicting relationships of all genomes, B; inset from A showing detailed relationships of P. kernoviae isolates. Chile 5 was not included in this analysis. [file MPP-20-423-s002.jpg]

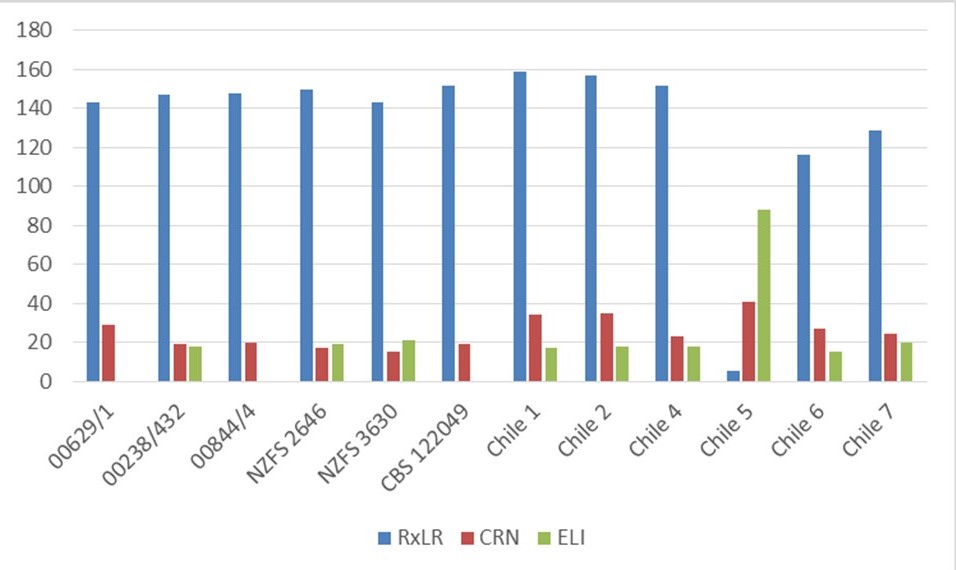

Supplement: Supplementary file 3 — Fig. S3 Numbers of predicted RxLR, CRN and elicitin genes from Phytophthora genomes. Elicitin gene numbers were not predicted from 0069/1, 00844/4 or CBS 122049. Genomes from strains 0069/1 and 00844/4 show a very high level of identity to that of 00238/432 (Studholme et al. unpublished), and strain CBS 122049 is also very similar to the other UK strains, hence 00238/432 was used solely to represent the UK strains. [file MPP-20-423-s003.jpg]
