# Supplementary material for: Genome sequencing of oomycete isolates from Chile supports the New Zealand origin of Phytophthora kernoviae and makes available the first Nothophytophthora sp. genome
Source: Mol Plant Pathol. 2018 Dec 5;20(3):423–31. doi: 10.1111/mpp.12765 (PMC6637878; doi:10.1111/mpp.12765)
Supplement: Supplementary file 4 — Table S1 Repeat Content of Chilean Phytophthora kernoviae and Nothophytophthora genomes. [file MPP-20-423-s004.docx]

**Table S1. Repeat Content of Chilean *Phytophthora* *kernoviae* and *Nothophytophthora* genomes**

|  |  | | | | | | | | | | | |
| --- | --- | --- | --- | --- | --- | --- | --- | --- | --- | --- | --- | --- |
| **Repeat Type** | **Chile1** | **Chile2** | **Chile4** | **Chile6** | **Chile7** | **00629/1 (UK)** | **00238/432 (UK)** | **00844/4 (UK)** | **CBS 122049**  **(UK)** | **NZFS 2646**  **(NZ)** | **NZFS 3630**  **(NZ)** | **Chile5**  **(*Nothophytophthora*)** |
| Retro-elements | 2.77 | 2.68 | 2.02 | 1.87 | 2.19 | 1.25 | 1.27 | 1.24 | 1.46 | 1.87 | 1.84 | 2.90 |
| SINEs | 0.00 | 0.00 | 0.00 | 0.00 | 0.00 | 1.25 | 1.27 | 0.00 | 0.00 | 0.00 | 0.00 | 0.00 |
| LINEs | 0.54 | 0.56 | 0.52 | 0.58 | 0.64 | 0.39 | 0.40 | 0.39 | 0.44 | 0.52 | 0.54 | 0.34 |
| LTR elements | 4.97 | 4.57 | 3.49 | 3.11 | 3.67 | 2.07 | 2.11 | 2.07 | 1.44 | 3.18 | 3.10 | 5.38 |
| DNA transposon | 1.42 | 1.51 | 1.38 | 1.49 | 1.55 | 1.07 | 1.05 | 1.04 | 1.16 | 1.33 | 1.35 | 2.11 |
| Unclassified | 0.00 | 0.00 | 0.00 | 0.01 | 0.01 | 0.00 | 0.00 | 0.00 | 0.00 | 0.00 | 0.00 | 0.17 |
| Interspersed | 4.08 | 4.06 | 3.28 | 3.17 | 3.56 | 2.21 | 2.21 | 2.17 | 2.52 | 3.08 | 3.07 | 4.72 |
| Small RNA | 0.02 | 0.01 | 0.02 | 0.02 | 0.02 | 0.02 | 0.02 | 0.02 | 0.00 | 0.02 | 0.02 | 0.01 |
| Simple Repeats | 0.43 | 0.44 | 0.43 | 0.43 | 0.43 | 0.37 | 0.38 | 0.38 | 0.38 | 0.42 | 0.43 | 0.79 |
| Low Complexity | 0.04 | 0.05 | 0.04 | 0.04 | 0.04 | 0.04 | 0.04 | 0.04 | 0.04 | 0.05 | 0.04 | 0.06 |
| **Totals** | **14.27** | **13.88** | **11.18** | **10.72** | **12.11** | **7.42** | **7.48** | **6.11** | **5.98** | **8.60** | **8.55** | **16.48** |
