# Supplementary material for: Genome sequencing of oomycete isolates from Chile supports the New Zealand origin of Phytophthora kernoviae and makes available the first Nothophytophthora sp. genome
Source: Mol Plant Pathol. 2018 Dec 5;20(3):423–31. doi: 10.1111/mpp.12765 (PMC6637878; doi:10.1111/mpp.12765)
Supplement: Supplementary file 5 — Text S1 Configuration files for MAKER genome annotation. [file MPP-20-423-s005.docx]

Configuration files for MAKER genome annotation

Chile 1: maker_bopts.ctl

#-----BLAST and Exonerate Statistics Thresholds

blast_type=ncbi+ #set to 'ncbi+', 'ncbi' or 'wublast'

pcov_blastn=0.8 #Blastn Percent Coverage Threhold EST-Genome Alignments

pid_blastn=0.85 #Blastn Percent Identity Threshold EST-Genome Aligments

eval_blastn=1e-10 #Blastn eval cutoff

bit_blastn=40 #Blastn bit cutoff

depth_blastn=0 #Blastn depth cutoff (0 to disable cutoff)

pcov_blastx=0.5 #Blastx Percent Coverage Threhold Protein-Genome Alignments

pid_blastx=0.4 #Blastx Percent Identity Threshold Protein-Genome Aligments

eval_blastx=1e-06 #Blastx eval cutoff

bit_blastx=30 #Blastx bit cutoff

depth_blastx=0 #Blastx depth cutoff (0 to disable cutoff)

pcov_tblastx=0.8 #tBlastx Percent Coverage Threhold alt-EST-Genome Alignments

pid_tblastx=0.85 #tBlastx Percent Identity Threshold alt-EST-Genome Aligments

eval_tblastx=1e-10 #tBlastx eval cutoff

bit_tblastx=40 #tBlastx bit cutoff

depth_tblastx=0 #tBlastx depth cutoff (0 to disable cutoff)

pcov_rm_blastx=0.5 #Blastx Percent Coverage Threhold For Transposable Element Masking

pid_rm_blastx=0.4 #Blastx Percent Identity Threshold For Transposbale Element Masking

eval_rm_blastx=1e-06 #Blastx eval cutoff for transposable element masking

bit_rm_blastx=30 #Blastx bit cutoff for transposable element masking

ep_score_limit=20 #Exonerate protein percent of maximal score threshold

en_score_limit=20 #Exonerate nucleotide percent of maximal score threshold

Chile 1: maker_exe.ctl

#-----Location of Executables Used by MAKER/EVALUATOR

makeblastdb=/usr/bin/makeblastdb #location of NCBI+ makeblastdb executable

blastn=/usr/bin/blastn #location of NCBI+ blastn executable

blastx=/usr/bin/blastx #location of NCBI+ blastx executable

tblastx=/usr/bin/tblastx #location of NCBI+ tblastx executable

formatdb=/usr/bin/formatdb #location of NCBI formatdb executable

blastall=/usr/bin/blastall #location of NCBI blastall executable

xdformat= #location of WUBLAST xdformat executable

blasta= #location of WUBLAST blasta executable

RepeatMasker=/home/djs217/RepeatMasker4/RepeatMasker #location of RepeatMasker executable

exonerate=/usr/bin/exonerate #location of exonerate executable

#-----Ab-initio Gene Prediction Algorithms

snap= #location of snap executable

gmhmme3= #location of eukaryotic genemark executable

gmhmmp= #location of prokaryotic genemark executable

augustus=/usr/local/bin/augustus #location of augustus executable

fgenesh= #location of fgenesh executable

tRNAscan-SE=/usr/local/bin/tRNAscan-SE #location of trnascan executable

snoscan= #location of snoscan executable

#-----Other Algorithms

probuild= #location of probuild executable (required for genemark)

Chile 1: maker_opts.ctl

#-----Genome (these are always required)

genome=GCA_001712655.1_PkChile1v1.0_genomic.fna #genome sequence (fasta file or fasta embeded in GFF3 file)

organism_type=eukaryotic #eukaryotic or prokaryotic. Default is eukaryotic

#-----Re-annotation Using MAKER Derived GFF3

maker_gff= #MAKER derived GFF3 file

est_pass=0 #use ESTs in maker_gff: 1 = yes, 0 = no

altest_pass=0 #use alternate organism ESTs in maker_gff: 1 = yes, 0 = no

protein_pass=0 #use protein alignments in maker_gff: 1 = yes, 0 = no

rm_pass=0 #use repeats in maker_gff: 1 = yes, 0 = no

model_pass=0 #use gene models in maker_gff: 1 = yes, 0 = no

pred_pass=0 #use ab-initio predictions in maker_gff: 1 = yes, 0 = no

other_pass=0 #passthrough anyything else in maker_gff: 1 = yes, 0 = no

#-----EST Evidence (for best results provide a file for at least one)

est= #set of ESTs or assembled mRNA-seq in fasta format

altest= #EST/cDNA sequence file in fasta format from an alternate organism

est_gff= #aligned ESTs or mRNA-seq from an external GFF3 file

altest_gff= #aligned ESTs from a closly relate species in GFF3 format

#-----Protein Homology Evidence (for best results provide a file for at least one)

protein=/data/uniprot/uniprot_sprot.fasta #protein sequence file in fasta format (i.e. from mutiple oransisms)

protein_gff= #aligned protein homology evidence from an external GFF3 file

#-----Repeat Masking (leave values blank to skip repeat masking)

model_org=all #select a model organism for RepBase masking in RepeatMasker

rmlib= #provide an organism specific repeat library in fasta format for RepeatMasker

repeat_protein=/home/djs217/maker/data/te_proteins.fasta #provide a fasta file of transposable element proteins for RepeatRunner

rm_gff= #pre-identified repeat elements from an external GFF3 file

prok_rm=0 #forces MAKER to repeatmask prokaryotes (no reason to change this), 1 = yes, 0 = no

softmask=1 #use soft-masking rather than hard-masking in BLAST (i.e. seg and dust filtering)

#-----Gene Prediction

snaphmm= #SNAP HMM file

gmhmm= #GeneMark HMM file

augustus_species=phytophthora_kernoviae #Augustus gene prediction species model

fgenesh_par_file= #FGENESH parameter file

pred_gff= #ab-initio predictions from an external GFF3 file

model_gff= #annotated gene models from an external GFF3 file (annotation pass-through)

est2genome=0 #infer gene predictions directly from ESTs, 1 = yes, 0 = no

protein2genome=1 #infer predictions from protein homology, 1 = yes, 0 = no

trna=0 #find tRNAs with tRNAscan, 1 = yes, 0 = no

snoscan_rrna= #rRNA file to have Snoscan find snoRNAs

unmask=0 #also run ab-initio prediction programs on unmasked sequence, 1 = yes, 0 = no

#-----Other Annotation Feature Types (features MAKER doesn't recognize)

other_gff= #extra features to pass-through to final MAKER generated GFF3 file

#-----External Application Behavior Options

alt_peptide=C #amino acid used to replace non-standard amino acids in BLAST databases

cpus=1 #max number of cpus to use in BLAST and RepeatMasker (not for MPI, leave 1 when using MPI)

#-----MAKER Behavior Options

max_dna_len=100000 #length for dividing up contigs into chunks (increases/decreases memory usage)

min_contig=2000 #skip genome contigs below this length (under 10kb are often useless)

pred_flank=200 #flank for extending evidence clusters sent to gene predictors

pred_stats=0 #report AED and QI statistics for all predictions as well as models

AED_threshold=1 #Maximum Annotation Edit Distance allowed (bound by 0 and 1)

min_protein=0 #require at least this many amino acids in predicted proteins

alt_splice=0 #Take extra steps to try and find alternative splicing, 1 = yes, 0 = no

always_complete=0 #extra steps to force start and stop codons, 1 = yes, 0 = no

map_forward=0 #map names and attributes forward from old GFF3 genes, 1 = yes, 0 = no

keep_preds=1 #Concordance threshold to add unsupported gene prediction (bound by 0 and 1)

split_hit=10000 #length for the splitting of hits (expected max intron size for evidence alignments)

single_exon=0 #consider single exon EST evidence when generating annotations, 1 = yes, 0 = no

single_length=250 #min length required for single exon ESTs if 'single_exon is enabled'

correct_est_fusion=0 #limits use of ESTs in annotation to avoid fusion genes

tries=2 #number of times to try a contig if there is a failure for some reason

clean_try=0 #remove all data from previous run before retrying, 1 = yes, 0 = no

clean_up=0 #removes theVoid directory with individual analysis files, 1 = yes, 0 = no

TMP= #specify a directory other than the system default temporary directory for temporary files

Chile 2: maker_bopts.ctl

#-----BLAST and Exonerate Statistics Thresholds

blast_type=ncbi+ #set to 'ncbi+', 'ncbi' or 'wublast'

pcov_blastn=0.8 #Blastn Percent Coverage Threhold EST-Genome Alignments

pid_blastn=0.85 #Blastn Percent Identity Threshold EST-Genome Aligments

eval_blastn=1e-10 #Blastn eval cutoff

bit_blastn=40 #Blastn bit cutoff

depth_blastn=0 #Blastn depth cutoff (0 to disable cutoff)

pcov_blastx=0.5 #Blastx Percent Coverage Threhold Protein-Genome Alignments

pid_blastx=0.4 #Blastx Percent Identity Threshold Protein-Genome Aligments

eval_blastx=1e-06 #Blastx eval cutoff

bit_blastx=30 #Blastx bit cutoff

depth_blastx=0 #Blastx depth cutoff (0 to disable cutoff)

pcov_tblastx=0.8 #tBlastx Percent Coverage Threhold alt-EST-Genome Alignments

pid_tblastx=0.85 #tBlastx Percent Identity Threshold alt-EST-Genome Aligments

eval_tblastx=1e-10 #tBlastx eval cutoff

bit_tblastx=40 #tBlastx bit cutoff

depth_tblastx=0 #tBlastx depth cutoff (0 to disable cutoff)

pcov_rm_blastx=0.5 #Blastx Percent Coverage Threhold For Transposable Element Masking

pid_rm_blastx=0.4 #Blastx Percent Identity Threshold For Transposbale Element Masking

eval_rm_blastx=1e-06 #Blastx eval cutoff for transposable element masking

bit_rm_blastx=30 #Blastx bit cutoff for transposable element masking

ep_score_limit=20 #Exonerate protein percent of maximal score threshold

en_score_limit=20 #Exonerate nucleotide percent of maximal score threshold

Chile 2: maker_exe.ctl

#-----Location of Executables Used by MAKER/EVALUATOR

makeblastdb=/usr/bin/makeblastdb #location of NCBI+ makeblastdb executable

blastn=/usr/bin/blastn #location of NCBI+ blastn executable

blastx=/usr/bin/blastx #location of NCBI+ blastx executable

tblastx=/usr/bin/tblastx #location of NCBI+ tblastx executable

formatdb=/usr/bin/formatdb #location of NCBI formatdb executable

blastall=/usr/bin/blastall #location of NCBI blastall executable

xdformat= #location of WUBLAST xdformat executable

blasta= #location of WUBLAST blasta executable

RepeatMasker=/home/djs217/RepeatMasker4/RepeatMasker #location of RepeatMasker executable

exonerate=/usr/bin/exonerate #location of exonerate executable

#-----Ab-initio Gene Prediction Algorithms

snap= #location of snap executable

gmhmme3= #location of eukaryotic genemark executable

gmhmmp= #location of prokaryotic genemark executable

augustus=/usr/local/bin/augustus #location of augustus executable

fgenesh= #location of fgenesh executable

tRNAscan-SE=/usr/local/bin/tRNAscan-SE #location of trnascan executable

snoscan= #location of snoscan executable

#-----Other Algorithms

probuild= #location of probuild executable (required for genemark)

Chile 2: maker_opts.ctl

#-----Genome (these are always required)

genome=GCA_001707905.1_PkChile2v1.0_genomic.fna #genome sequence (fasta file or fasta embeded in GFF3 file)

organism_type=eukaryotic #eukaryotic or prokaryotic. Default is eukaryotic

#-----Re-annotation Using MAKER Derived GFF3

maker_gff= #MAKER derived GFF3 file

est_pass=0 #use ESTs in maker_gff: 1 = yes, 0 = no

altest_pass=0 #use alternate organism ESTs in maker_gff: 1 = yes, 0 = no

protein_pass=0 #use protein alignments in maker_gff: 1 = yes, 0 = no

rm_pass=0 #use repeats in maker_gff: 1 = yes, 0 = no

model_pass=0 #use gene models in maker_gff: 1 = yes, 0 = no

pred_pass=0 #use ab-initio predictions in maker_gff: 1 = yes, 0 = no

other_pass=0 #passthrough anyything else in maker_gff: 1 = yes, 0 = no

#-----EST Evidence (for best results provide a file for at least one)

est= #set of ESTs or assembled mRNA-seq in fasta format

altest= #EST/cDNA sequence file in fasta format from an alternate organism

est_gff= #aligned ESTs or mRNA-seq from an external GFF3 file

altest_gff= #aligned ESTs from a closly relate species in GFF3 format

#-----Protein Homology Evidence (for best results provide a file for at least one)

protein=/data/uniprot/uniprot_sprot.fasta #protein sequence file in fasta format (i.e. from mutiple oransisms)

protein_gff= #aligned protein homology evidence from an external GFF3 file

#-----Repeat Masking (leave values blank to skip repeat masking)

model_org=all #select a model organism for RepBase masking in RepeatMasker

rmlib= #provide an organism specific repeat library in fasta format for RepeatMasker

repeat_protein=/home/djs217/maker/data/te_proteins.fasta #provide a fasta file of transposable element proteins for RepeatRunner

rm_gff= #pre-identified repeat elements from an external GFF3 file

prok_rm=0 #forces MAKER to repeatmask prokaryotes (no reason to change this), 1 = yes, 0 = no

softmask=1 #use soft-masking rather than hard-masking in BLAST (i.e. seg and dust filtering)

#-----Gene Prediction

snaphmm= #SNAP HMM file

gmhmm= #GeneMark HMM file

augustus_species=phytophthora_kernoviae #Augustus gene prediction species model

fgenesh_par_file= #FGENESH parameter file

pred_gff= #ab-initio predictions from an external GFF3 file

model_gff= #annotated gene models from an external GFF3 file (annotation pass-through)

est2genome=0 #infer gene predictions directly from ESTs, 1 = yes, 0 = no

protein2genome=1 #infer predictions from protein homology, 1 = yes, 0 = no

trna=0 #find tRNAs with tRNAscan, 1 = yes, 0 = no

snoscan_rrna= #rRNA file to have Snoscan find snoRNAs

unmask=0 #also run ab-initio prediction programs on unmasked sequence, 1 = yes, 0 = no

#-----Other Annotation Feature Types (features MAKER doesn't recognize)

other_gff= #extra features to pass-through to final MAKER generated GFF3 file

#-----External Application Behavior Options

alt_peptide=C #amino acid used to replace non-standard amino acids in BLAST databases

cpus=1 #max number of cpus to use in BLAST and RepeatMasker (not for MPI, leave 1 when using MPI)

#-----MAKER Behavior Options

max_dna_len=100000 #length for dividing up contigs into chunks (increases/decreases memory usage)

min_contig=2000 #skip genome contigs below this length (under 10kb are often useless)

pred_flank=200 #flank for extending evidence clusters sent to gene predictors

pred_stats=0 #report AED and QI statistics for all predictions as well as models

AED_threshold=1 #Maximum Annotation Edit Distance allowed (bound by 0 and 1)

min_protein=0 #require at least this many amino acids in predicted proteins

alt_splice=0 #Take extra steps to try and find alternative splicing, 1 = yes, 0 = no

always_complete=0 #extra steps to force start and stop codons, 1 = yes, 0 = no

map_forward=0 #map names and attributes forward from old GFF3 genes, 1 = yes, 0 = no

keep_preds=1 #Concordance threshold to add unsupported gene prediction (bound by 0 and 1)

split_hit=10000 #length for the splitting of hits (expected max intron size for evidence alignments)

single_exon=0 #consider single exon EST evidence when generating annotations, 1 = yes, 0 = no

single_length=250 #min length required for single exon ESTs if 'single_exon is enabled'

correct_est_fusion=0 #limits use of ESTs in annotation to avoid fusion genes

tries=2 #number of times to try a contig if there is a failure for some reason

clean_try=0 #remove all data from previous run before retrying, 1 = yes, 0 = no

clean_up=0 #removes theVoid directory with individual analysis files, 1 = yes, 0 = no

TMP= #specify a directory other than the system default temporary directory for temporary files

Chile 4: maker_bopts.ctl

#-----BLAST and Exonerate Statistics Thresholds

blast_type=ncbi+ #set to 'ncbi+', 'ncbi' or 'wublast'

pcov_blastn=0.8 #Blastn Percent Coverage Threhold EST-Genome Alignments

pid_blastn=0.85 #Blastn Percent Identity Threshold EST-Genome Aligments

eval_blastn=1e-10 #Blastn eval cutoff

bit_blastn=40 #Blastn bit cutoff

depth_blastn=0 #Blastn depth cutoff (0 to disable cutoff)

pcov_blastx=0.5 #Blastx Percent Coverage Threhold Protein-Genome Alignments

pid_blastx=0.4 #Blastx Percent Identity Threshold Protein-Genome Aligments

eval_blastx=1e-06 #Blastx eval cutoff

bit_blastx=30 #Blastx bit cutoff

depth_blastx=0 #Blastx depth cutoff (0 to disable cutoff)

pcov_tblastx=0.8 #tBlastx Percent Coverage Threhold alt-EST-Genome Alignments

pid_tblastx=0.85 #tBlastx Percent Identity Threshold alt-EST-Genome Aligments

eval_tblastx=1e-10 #tBlastx eval cutoff

bit_tblastx=40 #tBlastx bit cutoff

depth_tblastx=0 #tBlastx depth cutoff (0 to disable cutoff)

pcov_rm_blastx=0.5 #Blastx Percent Coverage Threhold For Transposable Element Masking

pid_rm_blastx=0.4 #Blastx Percent Identity Threshold For Transposbale Element Masking

eval_rm_blastx=1e-06 #Blastx eval cutoff for transposable element masking

bit_rm_blastx=30 #Blastx bit cutoff for transposable element masking

ep_score_limit=20 #Exonerate protein percent of maximal score threshold

en_score_limit=20 #Exonerate nucleotide percent of maximal score threshold

Chile 4: maker_exe.ctl

#-----Location of Executables Used by MAKER/EVALUATOR

makeblastdb=/usr/bin/makeblastdb #location of NCBI+ makeblastdb executable

blastn=/usr/bin/blastn #location of NCBI+ blastn executable

blastx=/usr/bin/blastx #location of NCBI+ blastx executable

tblastx=/usr/bin/tblastx #location of NCBI+ tblastx executable

formatdb=/usr/bin/formatdb #location of NCBI formatdb executable

blastall=/usr/bin/blastall #location of NCBI blastall executable

xdformat= #location of WUBLAST xdformat executable

blasta= #location of WUBLAST blasta executable

RepeatMasker=/home/djs217/RepeatMasker4/RepeatMasker #location of RepeatMasker executable

exonerate=/usr/bin/exonerate #location of exonerate executable

#-----Ab-initio Gene Prediction Algorithms

snap= #location of snap executable

gmhmme3= #location of eukaryotic genemark executable

gmhmmp= #location of prokaryotic genemark executable

augustus=/usr/local/bin/augustus #location of augustus executable

fgenesh= #location of fgenesh executable

tRNAscan-SE=/usr/local/bin/tRNAscan-SE #location of trnascan executable

snoscan= #location of snoscan executable

#-----Other Algorithms

probuild= #location of probuild executable (required for genemark)

Chile 4: maker_opts.ctl

#-----Genome (these are always required)

genome=GCA_001712715.1_PkChile4v1.0_genomic.fna #genome sequence (fasta file or fasta embeded in GFF3 file)

organism_type=eukaryotic #eukaryotic or prokaryotic. Default is eukaryotic

#-----Re-annotation Using MAKER Derived GFF3

maker_gff= #MAKER derived GFF3 file

est_pass=0 #use ESTs in maker_gff: 1 = yes, 0 = no

altest_pass=0 #use alternate organism ESTs in maker_gff: 1 = yes, 0 = no

protein_pass=0 #use protein alignments in maker_gff: 1 = yes, 0 = no

rm_pass=0 #use repeats in maker_gff: 1 = yes, 0 = no

model_pass=0 #use gene models in maker_gff: 1 = yes, 0 = no

pred_pass=0 #use ab-initio predictions in maker_gff: 1 = yes, 0 = no

other_pass=0 #passthrough anyything else in maker_gff: 1 = yes, 0 = no

#-----EST Evidence (for best results provide a file for at least one)

est= #set of ESTs or assembled mRNA-seq in fasta format

altest= #EST/cDNA sequence file in fasta format from an alternate organism

est_gff= #aligned ESTs or mRNA-seq from an external GFF3 file

altest_gff= #aligned ESTs from a closly relate species in GFF3 format

#-----Protein Homology Evidence (for best results provide a file for at least one)

protein=/data/uniprot/uniprot_sprot.fasta #protein sequence file in fasta format (i.e. from mutiple oransisms)

protein_gff= #aligned protein homology evidence from an external GFF3 file

#-----Repeat Masking (leave values blank to skip repeat masking)

model_org=all #select a model organism for RepBase masking in RepeatMasker

rmlib= #provide an organism specific repeat library in fasta format for RepeatMasker

repeat_protein=/home/djs217/maker/data/te_proteins.fasta #provide a fasta file of transposable element proteins for RepeatRunner

rm_gff= #pre-identified repeat elements from an external GFF3 file

prok_rm=0 #forces MAKER to repeatmask prokaryotes (no reason to change this), 1 = yes, 0 = no

softmask=1 #use soft-masking rather than hard-masking in BLAST (i.e. seg and dust filtering)

#-----Gene Prediction

snaphmm= #SNAP HMM file

gmhmm= #GeneMark HMM file

augustus_species=phytophthora_kernoviae #Augustus gene prediction species model

fgenesh_par_file= #FGENESH parameter file

pred_gff= #ab-initio predictions from an external GFF3 file

model_gff= #annotated gene models from an external GFF3 file (annotation pass-through)

est2genome=0 #infer gene predictions directly from ESTs, 1 = yes, 0 = no

protein2genome=1 #infer predictions from protein homology, 1 = yes, 0 = no

trna=0 #find tRNAs with tRNAscan, 1 = yes, 0 = no

snoscan_rrna= #rRNA file to have Snoscan find snoRNAs

unmask=0 #also run ab-initio prediction programs on unmasked sequence, 1 = yes, 0 = no

#-----Other Annotation Feature Types (features MAKER doesn't recognize)

other_gff= #extra features to pass-through to final MAKER generated GFF3 file

#-----External Application Behavior Options

alt_peptide=C #amino acid used to replace non-standard amino acids in BLAST databases

cpus=1 #max number of cpus to use in BLAST and RepeatMasker (not for MPI, leave 1 when using MPI)

#-----MAKER Behavior Options

max_dna_len=100000 #length for dividing up contigs into chunks (increases/decreases memory usage)

min_contig=2000 #skip genome contigs below this length (under 10kb are often useless)

pred_flank=200 #flank for extending evidence clusters sent to gene predictors

pred_stats=0 #report AED and QI statistics for all predictions as well as models

AED_threshold=1 #Maximum Annotation Edit Distance allowed (bound by 0 and 1)

min_protein=0 #require at least this many amino acids in predicted proteins

alt_splice=0 #Take extra steps to try and find alternative splicing, 1 = yes, 0 = no

always_complete=0 #extra steps to force start and stop codons, 1 = yes, 0 = no

map_forward=0 #map names and attributes forward from old GFF3 genes, 1 = yes, 0 = no

keep_preds=1 #Concordance threshold to add unsupported gene prediction (bound by 0 and 1)

split_hit=10000 #length for the splitting of hits (expected max intron size for evidence alignments)

single_exon=0 #consider single exon EST evidence when generating annotations, 1 = yes, 0 = no

single_length=250 #min length required for single exon ESTs if 'single_exon is enabled'

correct_est_fusion=0 #limits use of ESTs in annotation to avoid fusion genes

tries=2 #number of times to try a contig if there is a failure for some reason

clean_try=0 #remove all data from previous run before retrying, 1 = yes, 0 = no

clean_up=0 #removes theVoid directory with individual analysis files, 1 = yes, 0 = no

TMP= #specify a directory other than the system default temporary directory for temporary files

Chile 6: maker_bopts.ctl

#-----BLAST and Exonerate Statistics Thresholds

blast_type=ncbi+ #set to 'ncbi+', 'ncbi' or 'wublast'

pcov_blastn=0.8 #Blastn Percent Coverage Threhold EST-Genome Alignments

pid_blastn=0.85 #Blastn Percent Identity Threshold EST-Genome Aligments

eval_blastn=1e-10 #Blastn eval cutoff

bit_blastn=40 #Blastn bit cutoff

depth_blastn=0 #Blastn depth cutoff (0 to disable cutoff)

pcov_blastx=0.5 #Blastx Percent Coverage Threhold Protein-Genome Alignments

pid_blastx=0.4 #Blastx Percent Identity Threshold Protein-Genome Aligments

eval_blastx=1e-06 #Blastx eval cutoff

bit_blastx=30 #Blastx bit cutoff

depth_blastx=0 #Blastx depth cutoff (0 to disable cutoff)

pcov_tblastx=0.8 #tBlastx Percent Coverage Threhold alt-EST-Genome Alignments

pid_tblastx=0.85 #tBlastx Percent Identity Threshold alt-EST-Genome Aligments

eval_tblastx=1e-10 #tBlastx eval cutoff

bit_tblastx=40 #tBlastx bit cutoff

depth_tblastx=0 #tBlastx depth cutoff (0 to disable cutoff)

pcov_rm_blastx=0.5 #Blastx Percent Coverage Threhold For Transposable Element Masking

pid_rm_blastx=0.4 #Blastx Percent Identity Threshold For Transposbale Element Masking

eval_rm_blastx=1e-06 #Blastx eval cutoff for transposable element masking

bit_rm_blastx=30 #Blastx bit cutoff for transposable element masking

ep_score_limit=20 #Exonerate protein percent of maximal score threshold

en_score_limit=20 #Exonerate nucleotide percent of maximal score threshold

Chile6: maker_exe.ctl

#-----Location of Executables Used by MAKER/EVALUATOR

makeblastdb=/usr/bin/makeblastdb #location of NCBI+ makeblastdb executable

blastn=/usr/bin/blastn #location of NCBI+ blastn executable

blastx=/usr/bin/blastx #location of NCBI+ blastx executable

tblastx=/usr/bin/tblastx #location of NCBI+ tblastx executable

formatdb=/usr/bin/formatdb #location of NCBI formatdb executable

blastall=/usr/bin/blastall #location of NCBI blastall executable

xdformat= #location of WUBLAST xdformat executable

blasta= #location of WUBLAST blasta executable

RepeatMasker=/home/djs217/RepeatMasker4/RepeatMasker #location of RepeatMasker executable

exonerate=/usr/bin/exonerate #location of exonerate executable

#-----Ab-initio Gene Prediction Algorithms

snap= #location of snap executable

gmhmme3= #location of eukaryotic genemark executable

gmhmmp= #location of prokaryotic genemark executable

augustus=/usr/local/bin/augustus #location of augustus executable

fgenesh= #location of fgenesh executable

tRNAscan-SE=/usr/local/bin/tRNAscan-SE #location of trnascan executable

snoscan= #location of snoscan executable

#-----Other Algorithms

probuild= #location of probuild executable (required for genemark)

Chile 6: maker_opts.ctl

#-----Genome (these are always required)

genome=GCA_001712705.1_PkChile4v6.0_genomic.fna #genome sequence (fasta file or fasta embeded in GFF3 file)

organism_type=eukaryotic #eukaryotic or prokaryotic. Default is eukaryotic

#-----Re-annotation Using MAKER Derived GFF3

maker_gff= #MAKER derived GFF3 file

est_pass=0 #use ESTs in maker_gff: 1 = yes, 0 = no

altest_pass=0 #use alternate organism ESTs in maker_gff: 1 = yes, 0 = no

protein_pass=0 #use protein alignments in maker_gff: 1 = yes, 0 = no

rm_pass=0 #use repeats in maker_gff: 1 = yes, 0 = no

model_pass=0 #use gene models in maker_gff: 1 = yes, 0 = no

pred_pass=0 #use ab-initio predictions in maker_gff: 1 = yes, 0 = no

other_pass=0 #passthrough anyything else in maker_gff: 1 = yes, 0 = no

#-----EST Evidence (for best results provide a file for at least one)

est= #set of ESTs or assembled mRNA-seq in fasta format

altest= #EST/cDNA sequence file in fasta format from an alternate organism

est_gff= #aligned ESTs or mRNA-seq from an external GFF3 file

altest_gff= #aligned ESTs from a closly relate species in GFF3 format

#-----Protein Homology Evidence (for best results provide a file for at least one)

protein=/data/uniprot/uniprot_sprot.fasta #protein sequence file in fasta format (i.e. from mutiple oransisms)

protein_gff= #aligned protein homology evidence from an external GFF3 file

#-----Repeat Masking (leave values blank to skip repeat masking)

model_org=all #select a model organism for RepBase masking in RepeatMasker

rmlib= #provide an organism specific repeat library in fasta format for RepeatMasker

repeat_protein=/home/djs217/maker/data/te_proteins.fasta #provide a fasta file of transposable element proteins for RepeatRunner

rm_gff= #pre-identified repeat elements from an external GFF3 file

prok_rm=0 #forces MAKER to repeatmask prokaryotes (no reason to change this), 1 = yes, 0 = no

softmask=1 #use soft-masking rather than hard-masking in BLAST (i.e. seg and dust filtering)

#-----Gene Prediction

snaphmm= #SNAP HMM file

gmhmm= #GeneMark HMM file

augustus_species=phytophthora_kernoviae #Augustus gene prediction species model

fgenesh_par_file= #FGENESH parameter file

pred_gff= #ab-initio predictions from an external GFF3 file

model_gff= #annotated gene models from an external GFF3 file (annotation pass-through)

est2genome=0 #infer gene predictions directly from ESTs, 1 = yes, 0 = no

protein2genome=1 #infer predictions from protein homology, 1 = yes, 0 = no

trna=0 #find tRNAs with tRNAscan, 1 = yes, 0 = no

snoscan_rrna= #rRNA file to have Snoscan find snoRNAs

unmask=0 #also run ab-initio prediction programs on unmasked sequence, 1 = yes, 0 = no

#-----Other Annotation Feature Types (features MAKER doesn't recognize)

other_gff= #extra features to pass-through to final MAKER generated GFF3 file

#-----External Application Behavior Options

alt_peptide=C #amino acid used to replace non-standard amino acids in BLAST databases

cpus=1 #max number of cpus to use in BLAST and RepeatMasker (not for MPI, leave 1 when using MPI)

#-----MAKER Behavior Options

max_dna_len=100000 #length for dividing up contigs into chunks (increases/decreases memory usage)

min_contig=2000 #skip genome contigs below this length (under 10kb are often useless)

pred_flank=200 #flank for extending evidence clusters sent to gene predictors

pred_stats=0 #report AED and QI statistics for all predictions as well as models

AED_threshold=1 #Maximum Annotation Edit Distance allowed (bound by 0 and 1)

min_protein=0 #require at least this many amino acids in predicted proteins

alt_splice=0 #Take extra steps to try and find alternative splicing, 1 = yes, 0 = no

always_complete=0 #extra steps to force start and stop codons, 1 = yes, 0 = no

map_forward=0 #map names and attributes forward from old GFF3 genes, 1 = yes, 0 = no

keep_preds=1 #Concordance threshold to add unsupported gene prediction (bound by 0 and 1)

split_hit=10000 #length for the splitting of hits (expected max intron size for evidence alignments)

single_exon=0 #consider single exon EST evidence when generating annotations, 1 = yes, 0 = no

single_length=250 #min length required for single exon ESTs if 'single_exon is enabled'

correct_est_fusion=0 #limits use of ESTs in annotation to avoid fusion genes

tries=2 #number of times to try a contig if there is a failure for some reason

clean_try=0 #remove all data from previous run before retrying, 1 = yes, 0 = no

clean_up=0 #removes theVoid directory with individual analysis files, 1 = yes, 0 = no

TMP= #specify a directory other than the system default temporary directory for temporary files

Chile 7: maker_bopts.ctl

#-----BLAST and Exonerate Statistics Thresholds

blast_type=ncbi+ #set to 'ncbi+', 'ncbi' or 'wublast'

pcov_blastn=0.8 #Blastn Percent Coverage Threhold EST-Genome Alignments

pid_blastn=0.85 #Blastn Percent Identity Threshold EST-Genome Aligments

eval_blastn=1e-10 #Blastn eval cutoff

bit_blastn=40 #Blastn bit cutoff

depth_blastn=0 #Blastn depth cutoff (0 to disable cutoff)

pcov_blastx=0.5 #Blastx Percent Coverage Threhold Protein-Genome Alignments

pid_blastx=0.4 #Blastx Percent Identity Threshold Protein-Genome Aligments

eval_blastx=1e-06 #Blastx eval cutoff

bit_blastx=30 #Blastx bit cutoff

depth_blastx=0 #Blastx depth cutoff (0 to disable cutoff)

pcov_tblastx=0.8 #tBlastx Percent Coverage Threhold alt-EST-Genome Alignments

pid_tblastx=0.85 #tBlastx Percent Identity Threshold alt-EST-Genome Aligments

eval_tblastx=1e-10 #tBlastx eval cutoff

bit_tblastx=40 #tBlastx bit cutoff

depth_tblastx=0 #tBlastx depth cutoff (0 to disable cutoff)

pcov_rm_blastx=0.5 #Blastx Percent Coverage Threhold For Transposable Element Masking

pid_rm_blastx=0.4 #Blastx Percent Identity Threshold For Transposbale Element Masking

eval_rm_blastx=1e-06 #Blastx eval cutoff for transposable element masking

bit_rm_blastx=30 #Blastx bit cutoff for transposable element masking

ep_score_limit=20 #Exonerate protein percent of maximal score threshold

en_score_limit=20 #Exonerate nucleotide percent of maximal score threshold

Chile 7: maker_exe.ctl

#-----Location of Executables Used by MAKER/EVALUATOR

makeblastdb=/usr/bin/makeblastdb #location of NCBI+ makeblastdb executable

blastn=/usr/bin/blastn #location of NCBI+ blastn executable

blastx=/usr/bin/blastx #location of NCBI+ blastx executable

tblastx=/usr/bin/tblastx #location of NCBI+ tblastx executable

formatdb=/usr/bin/formatdb #location of NCBI formatdb executable

blastall=/usr/bin/blastall #location of NCBI blastall executable

xdformat= #location of WUBLAST xdformat executable

blasta= #location of WUBLAST blasta executable

RepeatMasker=/home/djs217/RepeatMasker4/RepeatMasker #location of RepeatMasker executable

exonerate=/usr/bin/exonerate #location of exonerate executable

#-----Ab-initio Gene Prediction Algorithms

snap= #location of snap executable

gmhmme3= #location of eukaryotic genemark executable

gmhmmp= #location of prokaryotic genemark executable

augustus=/usr/local/bin/augustus #location of augustus executable

fgenesh= #location of fgenesh executable

tRNAscan-SE=/usr/local/bin/tRNAscan-SE #location of trnascan executable

snoscan= #location of snoscan executable

#-----Other Algorithms

probuild= #location of probuild executable (required for genemark)

Chile 7: maker_opts.ctl

#-----Genome (these are always required)

genome=GCA_001712645.1_PkChile7v1.0_genomic.fna #genome sequence (fasta file or fasta embeded in GFF3 file)

organism_type=eukaryotic #eukaryotic or prokaryotic. Default is eukaryotic

#-----Re-annotation Using MAKER Derived GFF3

maker_gff= #MAKER derived GFF3 file

est_pass=0 #use ESTs in maker_gff: 1 = yes, 0 = no

altest_pass=0 #use alternate organism ESTs in maker_gff: 1 = yes, 0 = no

protein_pass=0 #use protein alignments in maker_gff: 1 = yes, 0 = no

rm_pass=0 #use repeats in maker_gff: 1 = yes, 0 = no

model_pass=0 #use gene models in maker_gff: 1 = yes, 0 = no

pred_pass=0 #use ab-initio predictions in maker_gff: 1 = yes, 0 = no

other_pass=0 #passthrough anyything else in maker_gff: 1 = yes, 0 = no

#-----EST Evidence (for best results provide a file for at least one)

est= #set of ESTs or assembled mRNA-seq in fasta format

altest= #EST/cDNA sequence file in fasta format from an alternate organism

est_gff= #aligned ESTs or mRNA-seq from an external GFF3 file

altest_gff= #aligned ESTs from a closly relate species in GFF3 format

#-----Protein Homology Evidence (for best results provide a file for at least one)

protein=/data/uniprot/uniprot_sprot.fasta #protein sequence file in fasta format (i.e. from mutiple oransisms)

protein_gff= #aligned protein homology evidence from an external GFF3 file

#-----Repeat Masking (leave values blank to skip repeat masking)

model_org=all #select a model organism for RepBase masking in RepeatMasker

rmlib= #provide an organism specific repeat library in fasta format for RepeatMasker

repeat_protein=/home/djs217/maker/data/te_proteins.fasta #provide a fasta file of transposable element proteins for RepeatRunner

rm_gff= #pre-identified repeat elements from an external GFF3 file

prok_rm=0 #forces MAKER to repeatmask prokaryotes (no reason to change this), 1 = yes, 0 = no

softmask=1 #use soft-masking rather than hard-masking in BLAST (i.e. seg and dust filtering)

#-----Gene Prediction

snaphmm= #SNAP HMM file

gmhmm= #GeneMark HMM file

augustus_species=phytophthora_kernoviae #Augustus gene prediction species model

fgenesh_par_file= #FGENESH parameter file

pred_gff= #ab-initio predictions from an external GFF3 file

model_gff= #annotated gene models from an external GFF3 file (annotation pass-through)

est2genome=0 #infer gene predictions directly from ESTs, 1 = yes, 0 = no

protein2genome=1 #infer predictions from protein homology, 1 = yes, 0 = no

trna=0 #find tRNAs with tRNAscan, 1 = yes, 0 = no

snoscan_rrna= #rRNA file to have Snoscan find snoRNAs

unmask=0 #also run ab-initio prediction programs on unmasked sequence, 1 = yes, 0 = no

#-----Other Annotation Feature Types (features MAKER doesn't recognize)

other_gff= #extra features to pass-through to final MAKER generated GFF3 file

#-----External Application Behavior Options

alt_peptide=C #amino acid used to replace non-standard amino acids in BLAST databases

cpus=1 #max number of cpus to use in BLAST and RepeatMasker (not for MPI, leave 1 when using MPI)

#-----MAKER Behavior Options

max_dna_len=100000 #length for dividing up contigs into chunks (increases/decreases memory usage)

min_contig=2000 #skip genome contigs below this length (under 10kb are often useless)

pred_flank=200 #flank for extending evidence clusters sent to gene predictors

pred_stats=0 #report AED and QI statistics for all predictions as well as models

AED_threshold=1 #Maximum Annotation Edit Distance allowed (bound by 0 and 1)

min_protein=0 #require at least this many amino acids in predicted proteins

alt_splice=0 #Take extra steps to try and find alternative splicing, 1 = yes, 0 = no

always_complete=0 #extra steps to force start and stop codons, 1 = yes, 0 = no

map_forward=0 #map names and attributes forward from old GFF3 genes, 1 = yes, 0 = no

keep_preds=1 #Concordance threshold to add unsupported gene prediction (bound by 0 and 1)

split_hit=10000 #length for the splitting of hits (expected max intron size for evidence alignments)

single_exon=0 #consider single exon EST evidence when generating annotations, 1 = yes, 0 = no

single_length=250 #min length required for single exon ESTs if 'single_exon is enabled'

correct_est_fusion=0 #limits use of ESTs in annotation to avoid fusion genes

tries=2 #number of times to try a contig if there is a failure for some reason

clean_try=0 #remove all data from previous run before retrying, 1 = yes, 0 = no

clean_up=0 #removes theVoid directory with individual analysis files, 1 = yes, 0 = no

TMP= #specify a directory other than the system default temporary directory for temporary files

**Perl script forestimating allele frequencies from SAMtols mpileup file**

**#!/usr/bin/perl -w**

**use strict;**

**use warnings;**

**my $usage = "Usage: $0 <samtools pileup file> <min depth>";**

**my $file = shift or die "$usage\n";**

**my $min_depth = shift or die "$usage\n";**

**open(FILE,"<$file") or die "Failed to open file '$file'\n$!\n";**

**warn "parsing file '$file'\n";**

**while (<FILE>) {**

**chomp;**

**my @fields = split /\t/;**

**my ($chromosome, $pos, $ref_base, $reads, $alignment ) = @fields;**

**#warn "@fields\n";**

**if ($ref_base =~ m/^[ACGTN]$/i and**

**$reads >= $min_depth and**

**$alignment =~ m/^[ACGT\^\$\,\.]+$/i ) {**

**#warn "Alignment: '$alignment'\n";**

**### Parse the alignment string ...**

**### Remove special symbols for read ends and starts**

**$alignment =~ s/\^.//gi;**

**$alignment =~ s/\$//gi;**

**### Count the dots and commas**

**my @agree_hits = ($alignment =~ m/[\,\.]/g );**

**#warn "Done counting dots and commas\n";**

**### Count the deletions**

**my @deletion;**

**while ($alignment =~ m/\-(\d+)/) {**

**my $n = $1;**

**if ( $alignment =~ m/(\-$n[ACGTNacgtn]{$n})/) {**

**my $deletion = $1;**

**#warn "\n$_\n$deletion\n";**

**push @deletion, $deletion;**

**$alignment =~ s/$deletion//;**

**}**

**}**

**#warn "Done counting deletions\n";**

**### Count the insertions**

**my @insertion;**

**while ($alignment =~ m/\+(\d+)/) {**

**my $n = $1;**

**if ( $alignment =~ m/(\+$n[ACGTNacgtn]{$n})/) {**

**my $insertion = "\\$1";**

**#warn "\n$_\n$insertion\n";**

**push @insertion, $insertion;**

**$alignment =~ s/$insertion//;**

**}**

**}**

**#warn "Done counting insertions\n";**

**### Count substitutions**

**my @a = ($alignment =~ m/a/gi );**

**my @c = ($alignment =~ m/c/gi );**

**my @g = ($alignment =~ m/g/gi );**

**my @t = ($alignment =~ m/t/gi );**

**my @n = ($alignment =~ m/n/gi );**

**my @star = ($alignment =~ m/\*/gi );**

**#warn "Done counting substitutions\n";**

**### Do a sanity check that everything adds up!**

**my $depth = @agree_hits + @a + @c + @g + @t + @n + @star;**

**my $length = length($alignment);**

**if ($depth == $reads) {**

**### Identify the change. Which is the most abundant base?**

**my %base2counts;**

**$base2counts{a} = @a;**

**$base2counts{c} = @c;**

**$base2counts{g} = @g;**

**$base2counts{t} = @t;**

**$base2counts{ lc($ref_base) } = @agree_hits;**

**#$base2counts{'*'} = @star;**

**#$base2counts{'ins'} = @insertion;**

**#$base2counts{'del'} = @deletion;**

**#$base2counts{'ref'} = @agree_hits;**

**my %counts2bases;**

**foreach my $base(keys %base2counts) {**

**$base = lc($base);**

**if ($base eq 'ref') {**

**$base = $ref_base;**

**}**

**my $count = $base2counts{$base};**

**$counts2bases{$count}{$base}++;**

**#warn "base '$base' => '$count'\n";**

**}**

**### Generate an ordered list of alleles, ordered by abundance**

**my @sorted_counts;**

**if (keys %counts2bases) {**

**@sorted_counts = sort {$b<=>$a} keys %counts2bases;**

**} else {**

**warn "No eligible bases found for position $chromosome: $pos\n";**

**}**

**my @ordered_bases;**

**foreach my $count (@sorted_counts) {**

**foreach my $base (keys %{ $counts2bases{$count} }) {**

**push @ordered_bases, $base;**

**}**

**}**

**my $most_abundant_base = shift @ordered_bases;**

**my $secondmost_abundant_base = shift @ordered_bases;**

**### What is the proportion of the first-most abundant base?**

**my $proportion_firstmost;**

**if (defined $most_abundant_base) {**

**$proportion_firstmost = $base2counts{$most_abundant_base} / $depth;**

**unless ($proportion_firstmost) {**

**warn "Most abundant base is $most_abundant_base: $proportion_firstmost\n";**

**warn "base2counts =\n";**

**foreach my $key (keys %base2counts) {**

**warn "\t$key => $base2counts{$key}\n";**

**}**

**die "Alignment = '$alignment'\n";**

**}**

**} else {**

**warn "At $chromosome $pos I cannot determine the most abundant base !\n\t$alignment\n";**

**$proportion_firstmost = 'NA';**

**}**

**### What is the proportion of the second-most abundant base?**

**my $proportion_secondmost;**

**if (defined $secondmost_abundant_base) {**

**$proportion_secondmost = $base2counts{$secondmost_abundant_base} / $depth;**

**} else {**

**$proportion_secondmost = 0;**

**#No second base!**

**}**

**#warn "$proportion_secondmost\n";**

**### Only print this if the first and second most abundant are A, C, G ot T**

**#warn "Two most abundant alleles are: $most_abundant_base and $secondmost_abundant_base\n";**

**if ($most_abundant_base =~ m/^[ACGT]$/i and $secondmost_abundant_base =~ m/^[ACGT]$/i) {**

**print "$chromosome\t$pos\t$proportion_firstmost\t$proportion_secondmost\t$depth\n";**

**}**

**} else {**

**warn "$_\nlength($alignment)=$length\n$depth != $reads\n";**

**}**

**}**

**}**

**R script for plotting frequency distributions of allele frequencies**

**### Load the files**

**srr870489 <-read.table("CBS122049.SRR870489.versus.GCA_000785735.2_NZFS2646v2_genomic.pileup.freq-first-and-second-most-abundant.csv", header=F)**

**srr870497 <-read.table("CBS122049.SRR870497.versus.GCA_000785735.2_NZFS2646v2_genomic.pileup.freq-first-and-second-most-abundant.csv", header=F)**

**chile1 <-read.table("chile-second.one.versus.GCA_000785735.2_NZFS2646v2_genomic.pileup.freq-first-and-second-most-abundant.csv", header=F)**

**chile2 <-read.table("chile-second.2.versus.GCA_000785735.2_NZFS2646v2_genomic.pileup.freq-first-and-second-most-abundant.csv", header=F)**

**chile4 <-read.table("chile-second.4.versus.GCA_000785735.2_NZFS2646v2_genomic.pileup.freq-first-and-second-most-abundant.csv", header=F)**

**chile6 <-read.table("chile-second.6.versus.GCA_000785735.2_NZFS2646v2_genomic.pileup.freq-first-and-second-most-abundant.csv", header=F)**

**chile7 <-read.table("chile-second.7.versus.GCA_000785735.2_NZFS2646v2_genomic.pileup.freq-first-and-second-most-abundant.csv", header=F)**

**pk238.431 <-read.table("Kerno238_431.versus.GCA_000785735.2_NZFS2646v2_genomic.pileup.freq-first-and-second-most-abundant.csv", header=F)**

**pk629.1 <-read.table("Kerno629_1.versus.GCA_000785735.2_NZFS2646v2_genomic.pileup.freq-first-and-second-most-abundant.csv", header=F)**

**pk844.4 <-read.table("Kerno844_4.versus.GCA_000785735.2_NZFS2646v2_genomic.pileup.freq-first-and-second-most-abundant.csv", header=F)**

**nzfs2646 <-read.table("NZFS2646.versus.GCA_000785735.2_NZFS2646v2_genomic.pileup.freq-first-and-second-most-abundant.csv", header=F)**

**nzfs3630 <-read.table("NZFS3630.versus.GCA_000785735.2_NZFS2646v2_genomic.pileup.freq-first-and-second-most-abundant.csv", header=F)**

**### Define function**

**plot.heterozygosity.histogram <- function(genome, genome.title, upper.lim, lower.lim, num.classes, min.depth, show.legend){**

**most.abundant <- genome$V3[**

**genome$V3 >= lower.lim &**

**genome$V3 <= upper.lim &**

**!is.na(genome$V3) &**

**genome$V5 >= min.depth ]**

**second.most.abundant <- genome$V4[**

**genome$V4 >= lower.lim &**

**genome$V4 <= upper.lim &**

**!is.na(genome$V4) &**

**genome$V5 >= min.depth ]**

**if (show.legend == T) {**

**hist(second.most.abundant,**

**#nclass=num.classes,**

**main = genome.title,**

**xlab='Proportion',**

**ylab='Probability density',**

**xlim=c(lower.lim, upper.lim),**

**col = rgb(1,0,0,0.4),**

**breaks=seq(lower.lim,upper.lim,l=num.classes),**

**freq=F**

**)**

**} else {**

**hist(second.most.abundant,**

**#nclass=num.classes,**

**main = genome.title,**

**xlab='',**

**ylab='',**

**xlim=c(lower.lim, upper.lim),**

**col = rgb(1,0,0,0.4),**

**breaks=seq(lower.lim,upper.lim,l=num.classes),**

**freq=F**

**)**

**}**

**hist(most.abundant,**

**add=T,**

**#nclass=num.classes,**

**col = rgb(0,0,1,0.4),**

**breaks=seq(lower.lim,upper.lim,l=num.classes),**

**freq=F**

**)**

**adjust <- 1**

**lines( density( most.abundant, adjust=adjust), col = rgb(0,0,1,0.4), lwd=4 )**

**lines( density( second.most.abundant, adjust=adjust), col = rgb(1,0,0,0.4), lwd=4 )**

**if (show.legend == T & F) {**

**legend('topleft',c('2nd-most abundant','Most abundant'),**

**fill = rgb(1:0,0,0:1,0.4), bty = 'n',**

**border = NA)**

**}**

**return(1)**

**}**

**### Do stuff**

**#upper.lim <- 0.90**

**#lower.lim <- 0.10**

**upper.lim <- 0.99**

**lower.lim <- 0.01**

**num.classes <- 21**

**min.depth <- 5**

**pdf('~/Dropbox/Pk.heterozygosity.pdf')**

**### Multiple graphs on the page**

**op<-par(mfrow=c(3,4))**

**plot.heterozygosity.histogram(chile1, "Chile 1", upper.lim, lower.lim, num.classes, min.depth, T)**

**plot.heterozygosity.histogram(chile2, "Chile 2", upper.lim, lower.lim, num.classes, min.depth, F)**

**plot.heterozygosity.histogram(chile4, "Chile 4", upper.lim, lower.lim, num.classes, min.depth, F)**

**plot.heterozygosity.histogram(chile6, "Chile 6", upper.lim, lower.lim, num.classes, min.depth, F)**

**plot.heterozygosity.histogram(chile7, "Chile 7", upper.lim, lower.lim, num.classes, min.depth, F)**

**plot.heterozygosity.histogram(pk238.431, "238/431", upper.lim, lower.lim, num.classes, min.depth, F)**

**plot.heterozygosity.histogram(pk629.1, "629/1", upper.lim, lower.lim, num.classes, min.depth, F)**

**plot.heterozygosity.histogram(pk844.4, "844/4", upper.lim, lower.lim, num.classes, min.depth, F)**

**plot.heterozygosity.histogram(nzfs2646, "NZFS 2646", upper.lim, lower.lim, num.classes, min.depth, F)**

**plot.heterozygosity.histogram(nzfs3630, "NZFS 3630", upper.lim, lower.lim, num.classes, min.depth, F)**

**#plot.heterozygosity.histogram(srr870489, "srr870489 HiSeq", upper.lim, lower.lim, num.classes, min.depth, F)**

**plot.heterozygosity.histogram(srr870497, "CBS 122049", upper.lim, lower.lim, num.classes, min.depth, F)**

**dev.off()**

**### Revert par**

**par(op)**
