# Supplementary material for: Genome sequencing of oomycete isolates from Chile supports the New Zealand origin of Phytophthora kernoviae and makes available the first Nothophytophthora sp. genome
Source: Mol Plant Pathol. 2018 Dec 5;20(3):423–31. doi: 10.1111/mpp.12765 (PMC6637878; doi:10.1111/mpp.12765)
Supplement: Supplementary file 6 — Text S2 Supplementary methods. (a) Phylogenetic analysis of COI region. (b) Analysis of genetic relationships using genome‐wide SNPs and SplitsTree. [file MPP-20-423-s006.docx]

**Supplementary methods**

**(a) Phylogenetic analysis of COI region**

Phylogenetic analysis was performed using the cytochrome oxidase I (COI). The phylogeny created using the COI gene was performed using the Maximum Likelihood method based on the Tamura-Nei model (Tamura and Nei 1993) using the PHYML plugin for Geneious (v10.2.2) (Kearse *et al.* 2012), and tree was edited in FigTree v1.4.3 (http://tree.bio.ed.ac.uk/software/figtree/). The phylogeny showed that there was genetic diversity within these isolates from Chile, but they were all most closely related to other *P. kernoviae* isolates (SUPPL Fig S1).

**(b) Analysis of genetic relationships using genome-wide SNPs and SplitsTree**

**SNPs were detected in the *P. kernoviae* genomes by aligning genomic sequence reads against the reference genome sequence of *P. kernoviae* strain 00238/432 (GenBank: GCA_000333075.2) using BWA mem, converting the resulting alignments into SAMtools mpileup format, and identifying sites where the aligned reads from at least one isolate showed a different nucleotide to the reference genome; these were considered as SNPs. Only sites that were unambiguously in data from all isolates were considered; if depth of coverage was less than 5x or if there was less than 95% consensus of the aligned reads in any of the isolates, then that position was denoted as ambiguous and excluded from further consideration. For each isolate, a sequence was generated, consisting of the consensus nucleotide at each of the SNP sites. The alignment of the SNP sequences was used as the input for tree construction** using the NeighborNet algorithm in SplitsTree (Huson & Bryant, 2006) to show the relationships between the UK, New Zealand and Chilean isolates of *P. kernoviae*.

**Kearse, M., R. Moir, A. Wilson, S. Stones-Havas, M. Cheung, S. Sturrock, S. Buxton, A. Cooper, S. Markowitz, C. Duran, T. Thierer, B. Ashton, P. Meintjes and A. Drummond** (2012). Geneious Basic: an integrated and extendable desktop software platform for the organization and analysis of sequence data. *Bioinformatics* **28**(12): 1647-1649.

**Tamura, K. and M. Nei** (1993). Estimation of the number of nucleotide substitutions in the control region of mitochondrial DNA in humans and chimpanzees. *Molecular Biology and Evolution* **10**(3): 512-526.
